# *Acinetobacter baumannii* can use multiple siderophores for iron acquisition, but only acinetobactin is required for virulence

**DOI:** 10.1371/journal.ppat.1008995

**Published:** 2020-10-19

**Authors:** Jessica R. Sheldon, Eric P. Skaar

**Affiliations:** 1 Department of Pathology, Microbiology, and Immunology, Vanderbilt University Medical Center, Nashville, Tennessee, United States of America; 2 Vanderbilt Institute for Infection, Immunology, and Inflammation, Vanderbilt University Medical Center, Nashville, Tennessee, United States of America; 3 Vanderbilt Institute of Chemical Biology, Vanderbilt University, Nashville, Tennessee, United States of America; Emory University School of Medicine, UNITED STATES

## Abstract

*Acinetobacter baumannii* is an emerging pathogen that poses a global health threat due to a lack of therapeutic options for treating drug-resistant strains. In addition to acquiring resistance to last-resort antibiotics, the success of *A*. *baumannii* is partially due to its ability to effectively compete with the host for essential metals. Iron is fundamental in shaping host-pathogen interactions, where the host restricts availability of this nutrient in an effort to curtail bacterial proliferation. To circumvent restriction, pathogens possess numerous mechanisms to obtain iron, including through the use of iron-scavenging siderophores. *A*. *baumannii* elaborates up to ten distinct siderophores, encoded from three different loci: acinetobactin and pre-acinetobactin (collectively, acinetobactin), baumannoferrins A and B, and fimsbactins A-F. The expression of multiple siderophores is common amongst bacterial pathogens and often linked to virulence, yet the collective contribution of these siderophores to *A*. *baumannii* survival and pathogenesis has not been investigated. Here we begin dissecting functional redundancy in the siderophore-based iron acquisition pathways of *A*. *baumannii*. Excess iron inhibits overall siderophore production by the bacterium, and the siderophore-associated loci are uniformly upregulated during iron restriction *in vitro* and *in vivo*. Further, disrupting all of the siderophore biosynthetic pathways is necessary to drastically reduce total siderophore production by *A*. *baumannii*, together suggesting a high degree of functional redundancy between the metabolites. By contrast, inactivation of acinetobactin biosynthesis alone impairs growth on human serum, transferrin, and lactoferrin, and severely attenuates survival of *A*. *baumannii* in a murine bacteremia model. These results suggest that whilst *A*. *baumannii* synthesizes multiple iron chelators, acinetobactin is critical to supporting growth of the pathogen on host iron sources. Given the acinetobactin locus is highly conserved and required for virulence of *A*. *baumannii*, designing therapeutics targeting the biosynthesis and/or transport of this siderophore may represent an effective means of combating this pathogen.

## Author summary

*Acinetobacter baumannii* is an emerging pathogen that is gaining notoriety as a major global health threat due to its extensive drug resistance. Accordingly, the World Health Organization has placed *A*. *baumannii* at the top of its list of bacteria urgently requiring research and development into novel therapeutic approaches, designating it a priority “critical” pathogen. Unfortunately, little is known about how *A*. *baumannii* causes infection. Iron is an essential nutrient to almost all forms of life and plays a key role in shaping host-pathogen interactions. *A*. *baumannii* secretes multiple low molecular weight iron chelators (siderophores) to help fulfill its need for the metal. Here we demonstrate that one of these siderophores, acinetobactin, is required for growth using host iron sources, whilst two others (baumannoferrin and fimsbactins) are dispensable to this function. Although all three siderophores are expressed under iron-restriction and in the infected host, disrupting acinetobactin production alone severely attenuates the survival of *A*. *baumannii* in a mouse model of bacteremia. Given the high degree of conservation of acinetobactin in *Acinetobacter* spp., and its essentiality to virulence, we propose that targeting production or uptake of this siderophore represents an attractive option for the development of novel therapeutics.

## Introduction

*Acinetobacter baumannii* is an opportunistic pathogen that is capable of causing a wide variety of diseases ranging from burn, wound, and urinary tract infections to more serious conditions such as ventilator-associated pneumonia and sepsis [1]. Initially regarded as a relatively innocuous nosocomial pathogen, *A*. *baumannii* has gained increased notoriety through the appearance of severe community-acquired infections [2–4], and concerningly, through the rapid emergence of multidrug-resistance [5–8]. As a result, the World Health Organization has placed *A*. *baumannii* at the top of its list of bacteria urgently requiring research and development into novel therapeutic approaches, designating it as a priority “critical” pathogen [9]. More recently, the Centers for Disease Control increased the threat level categorization of carbapenem-resistant *A*. *baumannii* from serious to urgent, due in part to a lack of drugs in the development pipeline [10]. Unfortunately, little is known about the factors that contribute to the survival and proliferation of *A*. *baumannii* within the host, and this knowledge is necessary for the informed design of new treatment options.

The success of *A*. *baumannii* as an emerging pathogen is not thought to be due to the evolution of traditional virulence factors, like toxins, but rather through a strategy referred to as “persist and resist” [11]. In addition to avoiding antibiotic-mediated killing, *A*. *baumannii* endures a wide variety of environmental insults including desiccation [12–14], oxidative stress [15–18], and micronutrient limitation [19–21]. Iron is one trace element that is essential to the vertebrate host and nearly all bacterial pathogens [22]. However, the concentration of free iron at the host-pathogen interface falls orders of magnitude below what is required to support microbial growth, due both to the formation of insoluble ferric oxyhydroxide precipitates under physiological conditions, and to the host maintaining stringent control over the availability of iron [23]. The withholding of metals, such as iron, to curtail bacterial growth in the host is a facet of the innate immune response referred to as “nutritional immunity” [24–26]. Host mechanisms of iron-withholding include maintaining it in complex with heme in hemoproteins, reducing ferroportin expression to limit its release from macrophages, and sequestering it within glycoproteins such as transferrin and lactoferrin, found predominantly in the blood and bodily secretions, respectively [27]. As such, accessing iron is perhaps one of the most formidable challenges faced by invading pathogens and is required for replication within the host.

**Fig 1:**
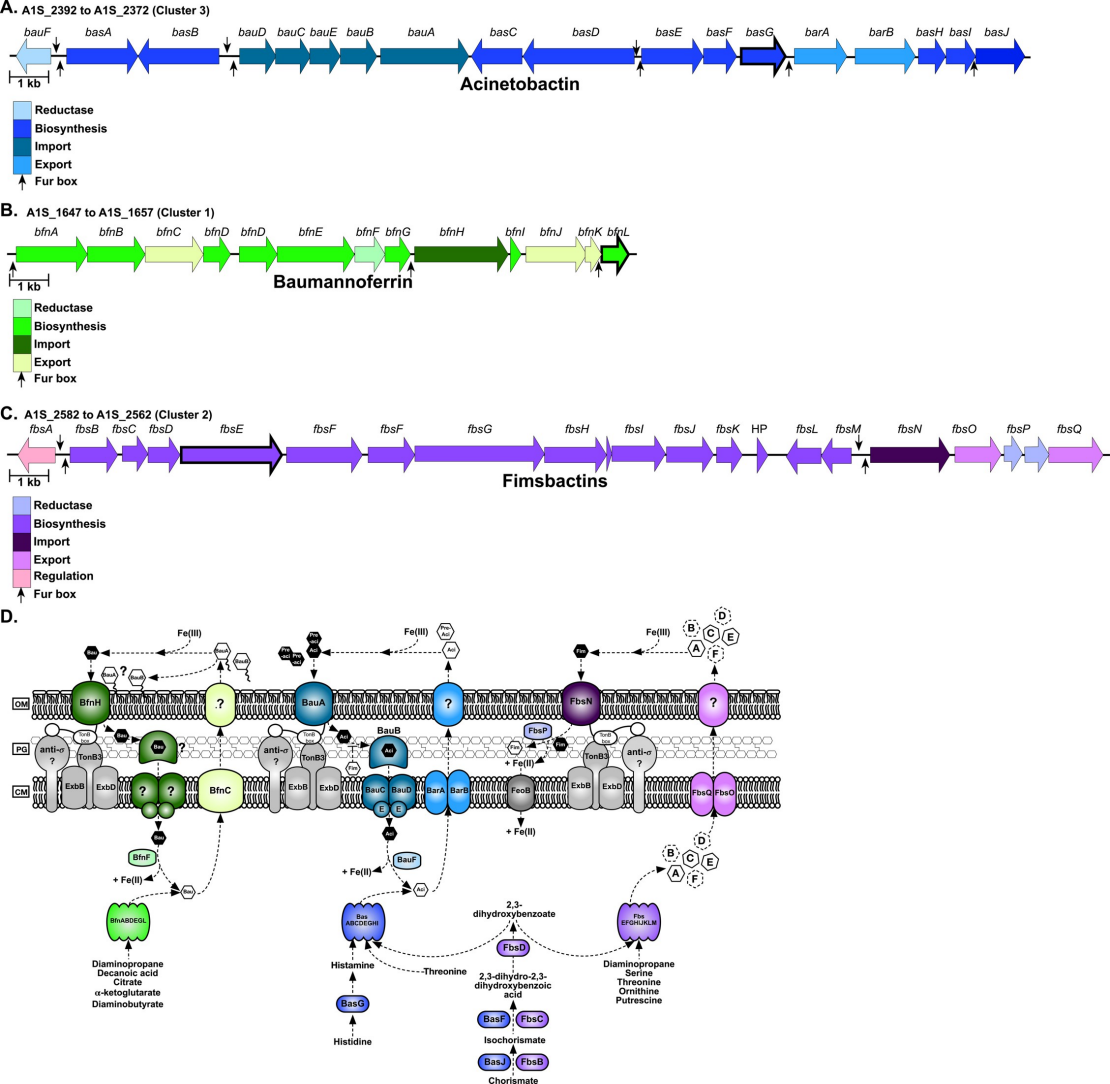
Genetic loci associated with siderophore biosynthesis and transport in *A*. *baumannii* ATCC 17978. Gene clusters for acinetobactin (A), baumannoferrin (B), and fimsbactins (C) are shown. Genes for the reductive release of iron from the siderophore, biosynthesis, import, export, and regulation are shown in the colors indicated in the corresponding legend. Putative Fur boxes are represented by black arrows, and the scale bar for each panel represents 1 kb. Key biosynthetic genes that were disrupted to elucidate siderophore function in this study are highlighted with thick black borders. A schematic highlighting the known and predicted siderophore biosynthetic and transport proteins in *A*. *baumannii* ATCC 17978, using the same color scheme as in A-C (D).

To overcome the barrier imposed by nutritional immunity, bacteria express a multitude of strategies to obtain metals. Broadly, the mechanisms of bacterial iron acquisition include capturing and extracting heme-iron from hemoproteins, expressing transporters for the uptake of free ferrous iron, and stealing iron from transferrin and lactoferrin by way of surface-bound receptor proteins and/or through secretion of small iron-binding molecules known as siderophores [22]. Many pathogens produce more than one type of siderophore, which are characterized based on the moieties used to coordinate ferric iron: catecholate, hydroxamate, and α-hydroxycarboxylate [28,29]. A fourth class includes siderophores possessing more than one of the aforementioned iron-coordinating groups and thus is referred to as “mixed-type” [28]. At least eight gene clusters have been identified to function in iron acquisition in *A*. *baumannii*, with the number of clusters present often differing between strains [30–32]. These clusters include a conserved ferrous iron acquisition system (*feoABC*) [33,34], two heme uptake loci (heme uptake cluster 1, and *hemO* cluster) [35], and five clusters involved in endogenous siderophore biosynthesis and utilization [30,31,36,37]. Of the siderophore biosynthetic clusters, one consists of two genes, *entA* and *entB*, which are used in the synthesis of 2,3-dihydroxybenzoic acid, a siderophore precursor molecule and weak iron chelator [31]. Another cluster, found only in *A*. *baumannii* strain 8399 is involved in the synthesis of an uncharacterized catecholate siderophore [36,37]. The remaining clusters have been more extensively investigated and encode for as many as ten structurally distinct siderophores, expressed from three loci and include acinetobactin and pre-acinetobactin [38–41], baumannoferrins A and B [42], and fimsbactins A through F [43] (Fig 1).

Acinetobactin is often referred to as being the major siderophore produced by *A*. *baumannii*, largely due to the high degree of conservation of the encoding locus amongst *Acinetobacter* clinical isolates [30,31,44]. The siderophore exists as two pH-dependent isomers, pre-acinetobactin which is released by the bacteria and is prevalent under acidic conditions, and acinetobactin which is favored under neutral and basic conditions [38,39,45]. All of the genes required for the biosynthesis (*basA-J*), efflux (*barAB*) and uptake (*bauA-E*) of pre-acinetobactin and acinetobactin (herein collectively referred to as acinetobactin) are encoded from the same locus, with the exception of an *entA* homologue, found elsewhere in the chromosome (Fig 1A and 1D; S1 and S3 Tables [41]). Expression of acinetobactin is required for virulence of *A*. *baumannii* strain ATCC 19606 in vertebrate and invertebrate models of infection [41,46]. It is important to note, however, that acinetobactin-mediated iron acquisition is the only high affinity iron uptake system that is functional in ATCC 19606 [41,47], and thus the importance of this siderophore in the context of multiple iron utilization systems is unknown. Indeed, the prevalence of at least two siderophore-encoding loci amongst clinical *A*. *baumannii* isolates highlights the importance of elucidating their individual contributions to infection [30].

In addition to acinetobactin, *in silico* analyses predict that most *A*. *baumannii* strains also express two hydroxamate-type siderophores, baumannoferrins A and B [30,31,42]. Twelve genes are thought to be involved in the biosynthesis (*bfnA*, *B*, *D*, *E*, *G*, *I* and *L*), transport (*bfnC*, *H*, *J*, and *K*), and utilization (*bfnF*) of the baumannoferrins and they are confined to a single locus (Fig 1B and 1D; S2 Table [42]). The *bfn* gene cluster bears homology to other loci associated with the nonribosomal peptide synthase (NRPS)-independent assembly of hydroxamate or carboxylate-type siderophores [42,48]. Purification and characterization of the baumannoferrins was performed using *A*. *baumannii* strain AYE [42], a commonly used clinical isolate which does not produce acinetobactin or other siderophores [41,49]. Structural elucidation revealed that baumannoferrins A and B differ only by one double-bond, but the significance of this is unknown [42]. Both baumannoferrins are able to chelate iron and facilitate growth of *A*. *baumannii* when supplied as an exogenous iron source [42,50]. Notably, *A*. *baumannii* strain AYE proliferates robustly under iron limitation [42], and is virulent in *Galleria mellonella* and mouse models of infection [51,52], suggesting that the expression of the baumannoferrins may play an important but underappreciated role in the survival and pathogenicity of *A*. *baumannii*.

Unlike with acinetobactin and baumannoferrin, whose loci are broadly represented in *A*. *baumannii* strains, the locus for fimsbactins biosynthesis and transport only appears to be present in a small fraction of sequenced *A*. *baumannii* isolates (~2%) [30,31,43,53]. Although the siderophores were initially isolated from the non-pathogenic species *Acinetobacter baylyi* [43], the fimsbactins cluster has been identified in pathogenic *Acinetobacter* isolates, including *A*. *baumannii* strain ATCC 17978 which is a commonly used laboratory strain that was initially isolated from a case of fatal meningitis [54], and AbPK1 which was identified as the causative agent of a deadly outbreak of pneumonia in sheep [53]. In the aforementioned species, the fimsbactins locus ((*fbsA-Q*) Fig 1C; S3 Table) is flanked by transposases, suggesting that it may have been acquired by horizontal gene transfer [31]. Like acinetobactin, the fimsbactins A-F are mixed catechol-hydroxamate type siderophores, where fimsbactin A represents the most abundant product of synthesis pathway, and fimsbactins B through F are likely biosynthetic intermediates or shunt products [43]. Given their structural similarity, it is perhaps not surprising that genes within the fimsbactins pathway (*fbsB*, *fbsC*, and *fbsH*) appear to be functionally redundant with those in the acinetobactin pathway ((*basJ*, *basF*, and *basE*, respectively) Fig 1D; S1 and S3 Tables [41]). Further, the sole *entA* homologue in *A*. *baumannii* strain ATCC 17978, required for both acinetobactin and fimsbactins production, appears to be located in the fimsbactins biosynthetic cluster (*fbsD*), and the siderophores are thought to compete for the same periplasmic binding protein [41,55]. The high degree of functional redundancy not only between acinetobactin and fimsbactins biosynthesis, but between the siderophore biosynthetic pathways overall in *A*. *baumannii* has confounded the ability to elucidate the individual function of the siderophores. Indeed, whilst purified fimsbactins A and B are capable of supporting the iron-dependent growth of *A*. *baumannii* [55,56], it is not known how endogenous expression of this siderophore influences survival of the pathogen *in vitro* or *in vivo*.

The expression of multiple pathways for the synthesis and utilization of siderophores is a common feature of bacterial pathogens, although the evolutionary reasons for having these seemingly redundant iron acquisition systems is not always known. With *A*. *baumannii*, the functional characterization of siderophores has historically been assessed using strains that inherently express only one siderophore, where determinations of overall importance to *A*. *baumannii* survival and pathogenesis can be difficult to make. In this study, we sought to address the complexity of *A*. *baumannii* siderophore-based iron acquisition systems by using a strain that encodes for acinetobactin, baumannoferrin, and fimsbactins [30,31], but does not utilize heme as an iron source [35]. A panel of *A*. *baumannii* ATCC 17978 mutants was generated where one, two, or all three siderophore biosynthetic pathways were genetically inactivated, such that the contributions of the siderophores could be assessed, both independently and in combination. The siderophores were found to be largely functionally redundant in their iron chelation ability *in vitro*, where disrupting all three biosynthetic pathways was required to substantially reduce total siderophore activity and to abolish growth under iron restriction. These findings were consistent with the observation that all three pathways are upregulated during iron-restriction *in vitro* and in the metal-restricted host. By contrast, disruption to acinetobactin biosynthesis alone was sufficient to attenuate growth on human serum, transferrin, or lactoferrin as a sole iron source. Strikingly, the acinetobactin biosynthetic mutant was severely attenuated for survival in the murine host, where reduced bacterial burdens were recovered from every major organ during infection. However, in the same model of murine bacteremia, the baumannoferrin and fimsbactins biosynthetic mutants did not exhibit a defect in survival, and a siderophore-deficient strain was no more attenuated for virulence than the acinetobactin mutant alone. Together these results suggest that whilst all three siderophores contribute to iron acquisition by *A*. *baumannii*, acinetobactin is the major siderophore required by this pathogen *in vivo* due to its ability to mobilize iron from host sources and its requirement for survival in a murine model of bacteremia.

## Results

### *Acinetobacter baumannii* siderophore biosynthesis and transport are upregulated in response to iron limitation

**Fig 2:**
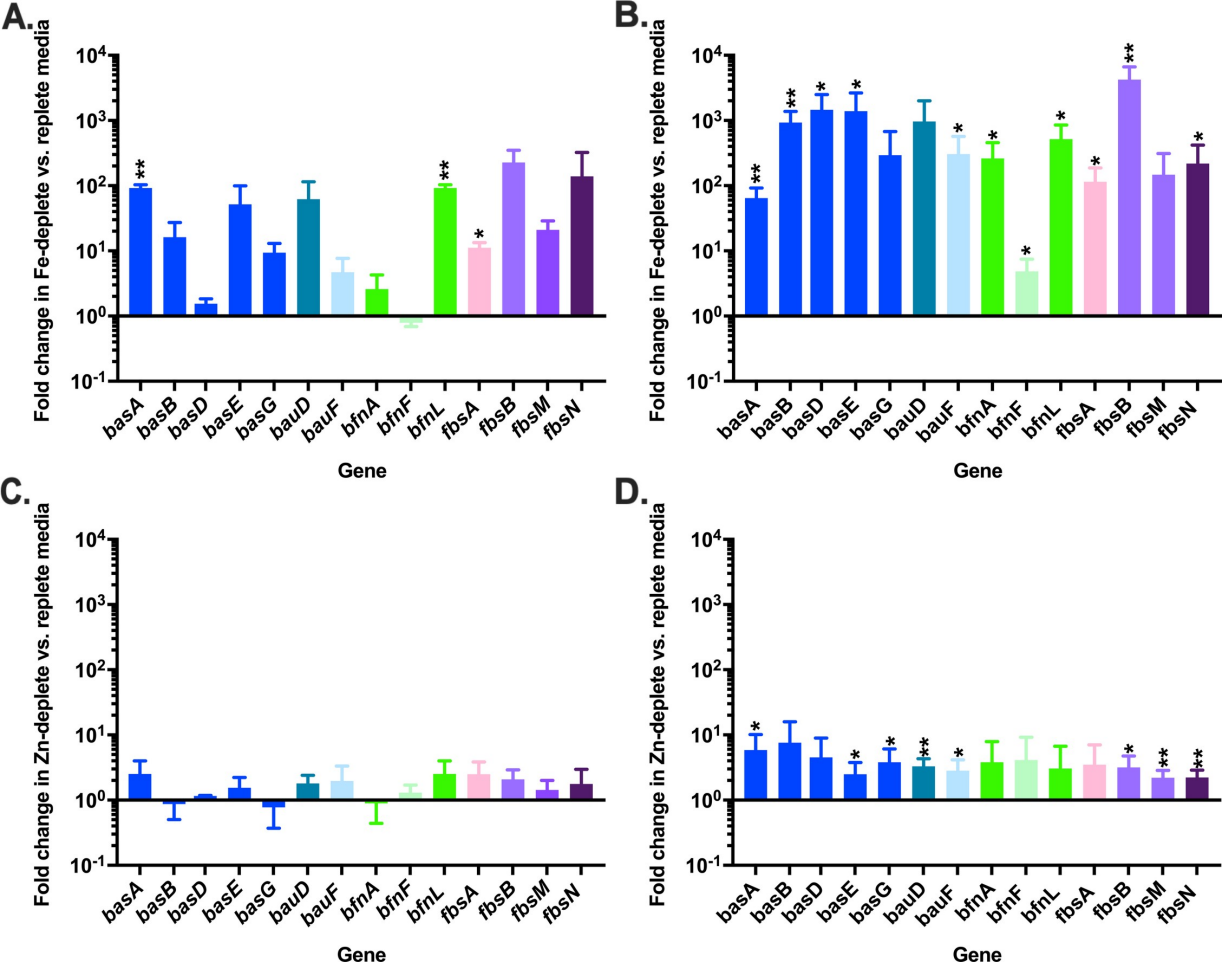
Siderophore biosynthetic and transport genes are upregulated in metal deplete conditions. WT *A*. *baumannii* was grown in metal-restricted media with or without the addition of exogenous iron or zinc. RNA was extracted and transcriptional changes in the expression of siderophore-associated genes were assessed by qRT-PCR in iron-deplete vs. replete conditions at 4 (A) and 12 h (B), and zinc-deplete vs. replete at 4 (C) and 12 (D) conditions, where expression was normalized to the expression of *rpoB*. * p < 0.05 and ** p < 0.01, as determined by Student’s *t* test relative to a hypothetical value of 1. Data are the means combined from two independent experiments, each with three biological replicates.

*A*. *baumannii* strain ATCC 17978 encodes for ten structurally distinct siderophores encoded from three different biosynthetic loci (Fig 1; S1, S2 and S3 Tables). We previously observed that expression of the loci encoding for the biosynthesis and transport of acinetobactin, baumannoferrin, and fimsbactins is upregulated in the presence of the multi-metal sequestering innate immune protein, calprotectin [57]. Given the apparent functional redundancies in the siderophore pathways of *A*. *baumannii*, we hypothesized that these systems are differentially regulated, thus allowing for expression in response to distinct environmental cues, such as differences in the availability of essential transition metals over time. To address this, wild-type (WT) *A*. *baumannii* was grown in metal-chelated Tris minimal succinate media (chelex-treated; cTMS) with and without the addition of exogenous iron or zinc. Following 4 or 12 hours (h) growth, RNA was extracted and the expression of various siderophore-associated transcripts from each of the three loci was assessed by quantitative reverse transcription PCR (qRT-PCR). Consistent with identification of putative Fur boxes upstream of many genes or operons within these loci (Fig 1A and 1B, and 1C and reference [31]), key biosynthetic, transport, and regulatory genes in all three siderophore-associated loci are strongly upregulated in iron-deplete versus replete conditions (Fig 2A and 2B). By contrast, and with the exception of *bfnF*, which encodes a putative oxidoreductase thought to release iron from the siderophore, the change in gene expression in zinc-deplete versus -replete conditions is 10 to 1000-fold lower than that observed with iron (Fig 2C and 2D), suggesting that the expression of genes encoding for siderophore biosynthesis and transport in *A*. *baumannii* is predominantly iron-regulated. Notably, gene expression under zinc limitation increased between 4 and 12 h, suggesting that zinc may play a role in the regulation of the siderophore-associated loci during prolonged metal restriction. To determine if these genes are directly regulated by the zinc uptake regulator, Zur, we performed qRT-PCR on a representative gene from each locus in WT *A*. *baumannii* and a Δ*zur* mutant [58]. Additionally, we assessed whether Zur can directly regulate *fur* by similarly looking at expression of this transcriptional regulator in the *zur* mutant. We found that although the expression of *fur* was unaltered in Δ*zur*, *basA* and *bfnA* were slightly but significantly upregulated in this background (S1 Fig). Together these results suggest that whilst Zur does not appear to directly regulate *fur* in *A*. *baumannii*, it may play a small but unappreciated role in the expression of known *fur*-regulated genes.

**Fig 3:**
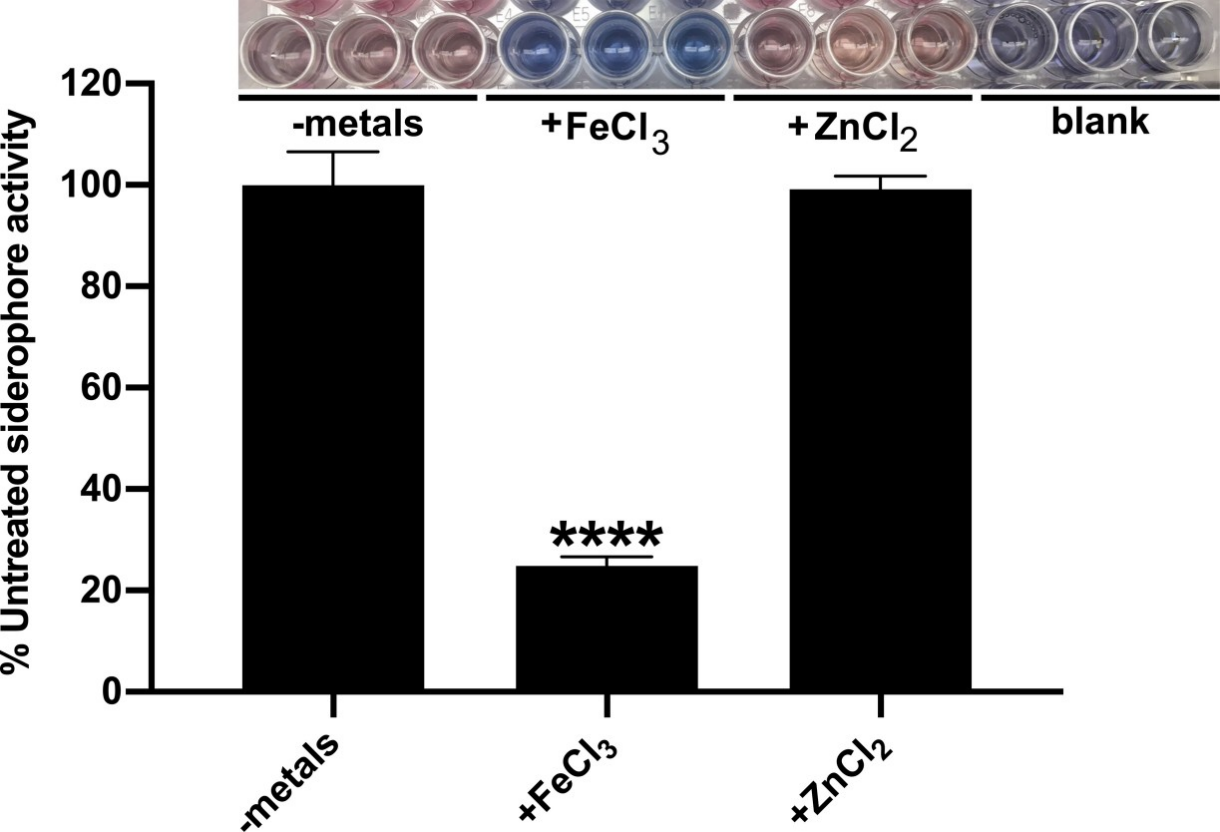
Iron, but not zinc, represses the overall siderophore activity of *A*. *baumannii*. Chrome Azurol S (CAS) assays to assess for total siderophore activity were performed on the spent culture supernatants of WT *A*. *baumannii* grown in cTMS media for 12 h with or without the addition of exogenous metals, as indicated. The colorimetric changes observed in the CAS assay are shown, where blue indicates an absence of siderophore activity and orange indicates that an iron chelator capable of mobilizing iron from CAS is present. Total siderophore activity is expressed as the percent activity of WT *A*. *baumannii* grown in the absence of exogenously added metals. **** p < 0.0001, as determined by one-way analysis of variance (ANOVA) with Dunnett’s multiple comparisons test. Data are the means of three biological replicates and are representative of three independent assays.

To determine if the transcriptional changes observed above translate to overall differences in siderophore production by *A*. *baumannii*, WT bacteria were grown in cTMS with and without the addition of exogenous metals. Following 12 h growth, assessment of siderophore activity in the *A*. *baumannii* supernatants was assessed by Chrome Azurol S (CAS) assay. CAS is an iron-binding dye where mobilization of iron from CAS to a chelator within the media can be detected through a colorimetric change of the dye from blue to orange [59,60]. Consistent with the impact of iron on gene transcription, the addition of iron to *A*. *baumannii* cultures strongly represses overall siderophore production by the bacteria (Fig 3), whereas the addition of zinc does not appear to impact this activity. These observations indicate that derepression of siderophore-associated genes during zinc limitation may not lead to increased siderophore production overall, and that acinetobactin, baumannoferrin, and fimsbactins biosynthesis and utilization in *A*. *baumannii* is primarily influenced by iron availability. It is possible, however, that the semi-quantitative nature of assessing total siderophore activity by CAS assay is not sensitive enough to detect subtle changes in siderophore production upon zinc limitation over time, as the accumulation of siderophores and/or siderophore biosynthetic enzymes may mask these effects.

### *A*. *baumannii* siderophores exhibit functional redundancy *in vitro*

To begin identifying the contribution of acinetobactin, baumannoferrin, and fimsbactins production to *A*. *baumannii* iron acquisition, key genes in each of the siderophore biosynthetic pathways were disrupted using recombineering [61]. The details of all strains employed in this study can be found in Materials and Methods, and the genes disrupted are highlighted in Fig 1. Genes targeted for inactivation were selected based on predicted function, as well as a lack of any potentially redundant homologues present elsewhere in the *A*. *baumannii* ATCC 17978 genome. The disrupted genes include *basG*, encoding a putative histidine decarboxylase predicted to synthesize histamine as an essential precursor molecule to acinetobactin biosynthesis [44,62], *bfnL*, encoding a putative acetyltransferase predicted to facilitate the conversion of N^3^-hydroxy-1,3-diaminopropane to N^3^-decanoyl-hydroxy-1,3-diaminopropane as an early step in baumannoferrin biosynthesis [42], and *fbsE*, encoding a putative nonribosomal peptide synthase (NRPS) and the first part of the multi-modular unit required to assemble fimsbactins [43].

**Fig 4:**
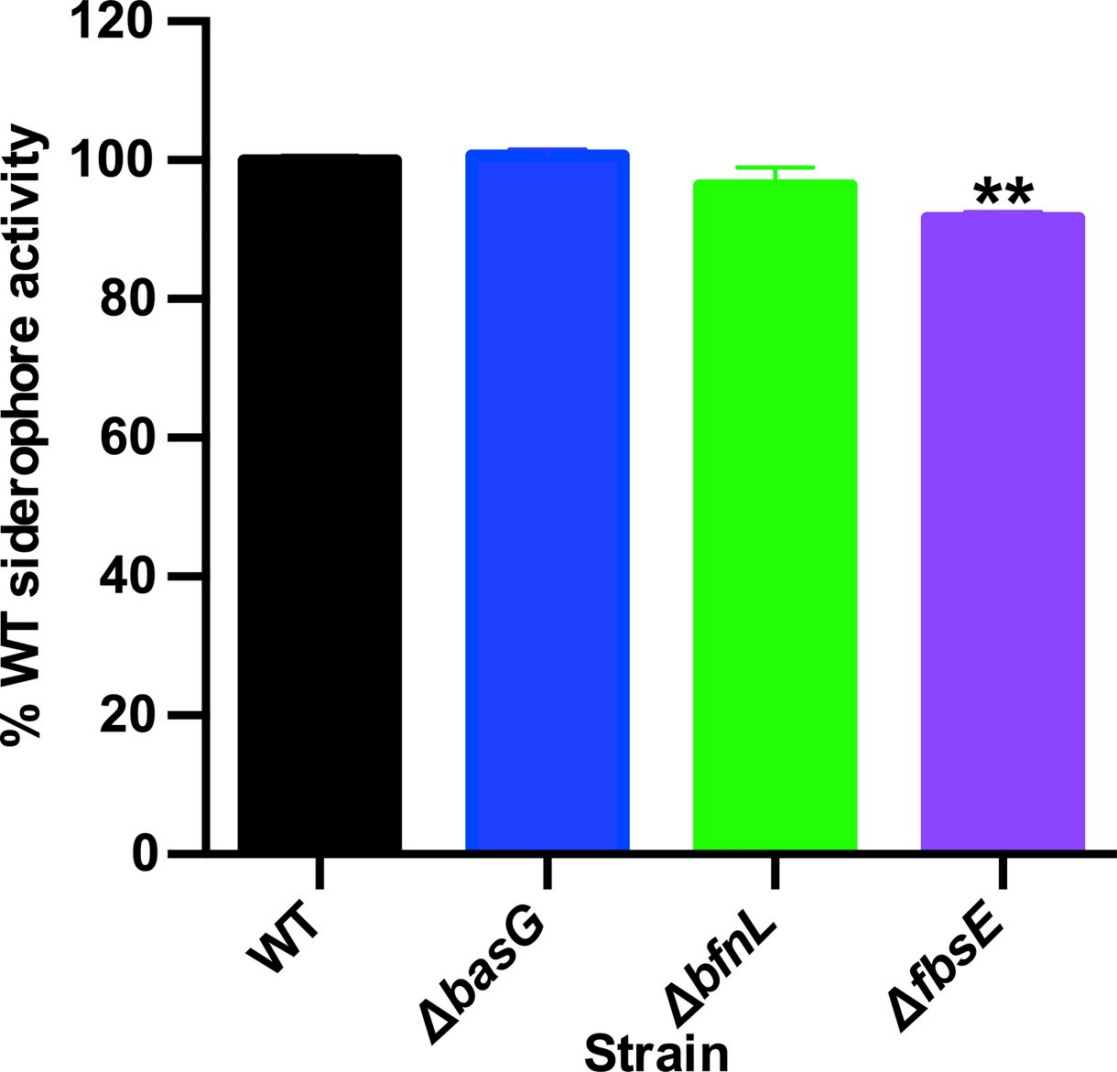
Disrupting a single siderophore biosynthetic pathway does not drastically impact overall siderophore activity in *A*. *baumannii*. Wild-type (WT) *A*. *baumannii* and its isogenic acinetobactin (Δ*basG*), baumannoferrin (Δ*bfnL)* and fimsbactins (Δ*fbsE*) biosynthetic mutants were grown for 12 h in cTMS, and CAS assays to assess overall siderophore activity were performed on the spent culture supernatants. Total siderophore activity is expressed as the percent activity of WT *A*. *baumannii*, where data are the means of three biological replicates and are representative of three independent assays. ** p < 0.01, as determined by one-way analysis ANOVA with Dunnett’s multiple comparisons test.

To determine if disruption to any one siderophore biosynthetic pathway impacts the ability of *A*. *baumannii* to chelate iron, WT *A*. *baumannii* and the strains inactivated for acinetobactin (Δ*basG*), baumannoferrin (Δ*bfnL*), or fimsbactins (Δ*fbsE*) biosynthesis were grown in cTMS for 12 h and CAS assays were performed on the culture supernatants. Aside from a small but reproducible reduction in siderophore activity in the Δ*fbsE* mutant, inactivating one siderophore pathway in *A*. *baumannii* does not drastically impact total siderophore activity (Fig 4). These results suggest that functional redundancy exists between the siderophore-based iron chelation activities of *A*. *baumannii in vitro*.

### Acinetobactin is required for maximal growth on host iron sources

**Fig 5:**
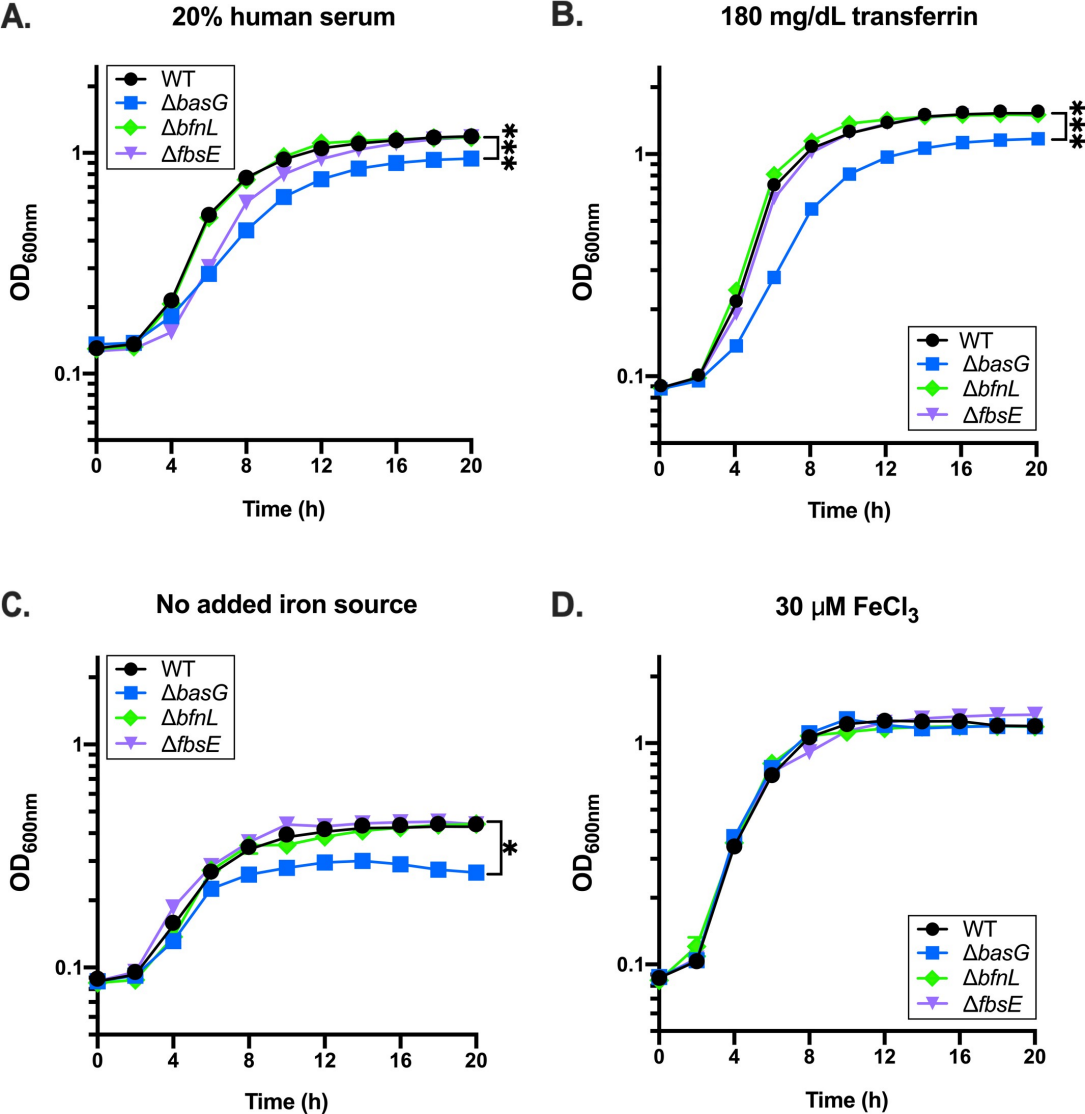
Acinetobactin biosynthetic mutants are impaired for growth under iron restriction. Wild-type (WT) *A*. *baumannii* and its isogenic acinetobactin (Δ*basG*), baumannoferrin (Δ*bfnL)* and fimsbactins (Δ*fbsE*) biosynthetic mutants were grown in cTMS media with 20% human serum (A), 180 mg/dL human transferrin (B), no added iron source (C), or 30 μM FeCl_3_ (D). Bacterial growth was assessed by determining the optical density at 600 nm (OD_600_), at the time points indicated. Data are representative of three independent experiments, and error bars represent the standard error of the mean. Where error bars are not visible, they are shorter than the height of the symbol. Statistical analysis is given for the endpoint growth, as performed by repeated measures two-way ANOVA, where *p < 0.05 and *** p < 0.001. Additional analyses of growth kinetics for these data can be found in S2 Fig.

In addition to sequestering small amounts of free ferric iron from the extracellular milieu, siderophores are capable of pirating iron from host glycoproteins such as transferrin and lactoferrin. To determine if acinetobactin, baumannoferrin, or fimsbactins are essential for iron acquisition from host iron sources, WT *A*. *baumannii* and the three siderophore biosynthetic mutants, Δ*basG*, Δ*bfnL*, and Δ*fbsE*, were grown in cTMS media with 20% human serum as the sole iron source. Bacterial growth was monitored by optical density (OD_6oonm_) over 20 hours. In contrast to the results of the CAS assay indicating that none of the mutations strongly impact overall siderophore production, the Δ*basG* mutant was found to be impaired for growth in serum relative to WT *A*. *baumannii* and the Δ*bfnL* and Δ*fbsE* mutants (Fig 5A). The defect in growth of Δ*basG* in serum is likely due to reduced acquisition of iron from transferrin, as this acinetobactin-deficient mutant is similarly impaired for growth on human transferrin as a sole iron source (Fig 5B). In addition to growing to a lower maximal OD_6oonm_ than WT when grown under iron restriction (Fig 5 and S2 Fig), further analysis of the growth kinetics of the *basG*-deficient strain revealed that that it also exhibits a statistically significant decrease in growth rate, and an increase in lag time under these conditions (S2 Fig). Although the Δ*fbsE* mutant exhibits a slight lag and decrease in growth rate in serum, it ultimately reaches an OD_6oonm_ comparable to WT and the Δ*bfnL* mutant (Fig 5A and S2 Fig). These effects were confirmed to be iron-dependent, as none of the strains are capable of robust growth in cTMS without an exogenous iron source (Fig 5C) but grow equivalently in the same media in the presence of excess free ferric iron (Fig 5D). The aforementioned phenotypes could be complemented by expression of *basG in trans* (S3 Fig), and are not specific to *A*. *baumannii* ATCC 17978, as similar results were observed for a *basG*-deficient strain of AB5075 (S4 Fig), a multidrug-resistant isolate that possesses the loci to express both acinetobactin and baumannoferrins [63]. Additionally, the *basG*-deficient strain also exhibited a similar growth defect when lactoferrin was provided as the sole iron source (S5 Fig). Together these findings show that iron is essential to the proliferation of *A*. *baumannii*, and that acinetobactin is required for maximal acquisition of iron from host sources such as serum transferrin, and lactoferrin.

### Expression of a single siderophore biosynthetic pathway is sufficient to promote WT siderophore activity

**Fig 6:**
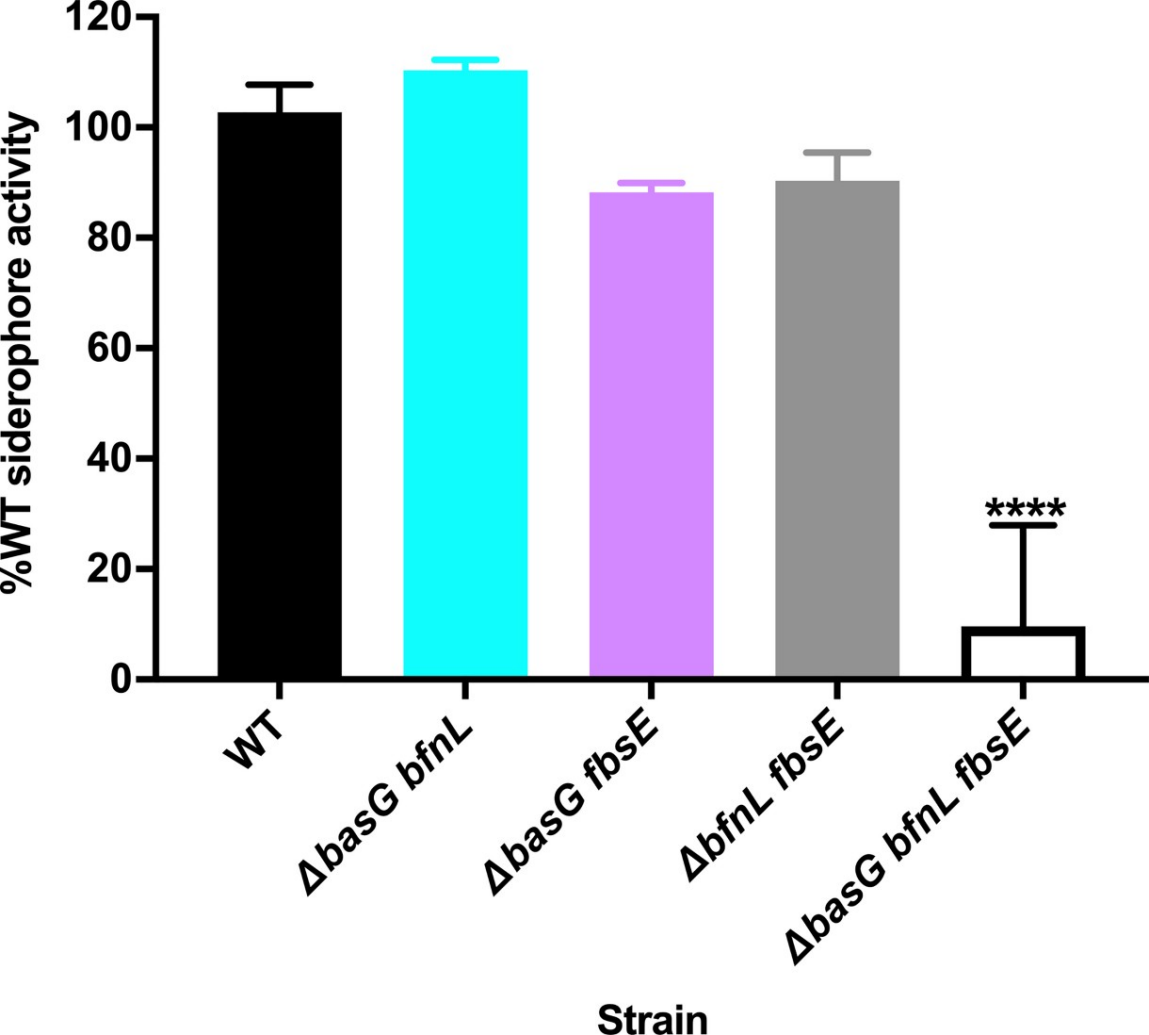
Disruption to all three siderophore biosynthetic pathways in *A*. *baumannii* is required to severely attenuate overall siderophore activity. Wild-type (WT) *A*. *baumannii* and its isogenic combinatorial mutants were grown for 12 h in cTMS, and CAS assays to assess overall siderophore production were performed on the spent culture supernatants. Total siderophore activity is expressed as the percent activity of WT *A*. *baumannii*, where data are the means of three biological replicates and are representative of three independent assays. **** p < 0.0001, as determined by one-way ANOVA with Dunnett’s multiple comparisons test.

To ascertain the role of each individual siderophore in *A*. *baumannii* iron acquisition, it was necessary to generate strains with only one siderophore biosynthetic pathway intact. To this end, double mutants were generated, such that a panel of strains capable of synthesizing only acinetobactin (Δ*bfnL fbsE*), baumannoferrin (Δ*basG fbsE*), or fimsbactins (Δ*basG bfnL*) was available. To assess the overall role of siderophore production on *A*. *baumannii* growth and pathogenesis, a strain with disruptions in each of the three biosynthetic pathways (Δ*basG bfnL fbsE*) was produced. With these strains in hand, the CAS assays were revisited, assessing overall siderophore activity in the culture supernatants of WT *A*. *baumannii* and its isogenic mutants grown in cTMS for 12 h. Interestingly, whilst the Δ*basG fbsE* and Δ*bfnL fbsE* mutants exhibited a small but reproducible reduction in overall siderophore activity relative to the WT, none of the double mutants were drastically impaired overall (Fig 6). In fact, the Δ*basG bfnL* mutant consistently exhibited increased CAS activity relative to the WT, suggesting that fimsbactins may be overexpressed in the absence of the other two siderophores, although the difference at 12 h was not statistically significant. We did find that genes within the fimsbactins-encoding locus are upregulated approximately 6.6 to 10-fold under the same conditions (S6 Fig), indicating that the loss of acinetobactin and baumannoferrin may induce further iron starvation in the bacteria leading to further transcriptional derepression of fimsbactins. In contrast to the robust siderophore production by the double mutants, when all three siderophore pathways were disrupted in the Δ*basG bfnL fbsE* mutant, siderophore activity was severely attenuated (Fig 6). Overall, these findings demonstrate that acinetobactin, baumannoferrin, and fimsbactins appear to be the predominant iron chelators produced by *A*. *baumannii*, and that expression of just one of these siderophores is sufficient to confer robust iron chelating activity *in vitro*.

### Siderophores are essential for *A*. *baumannii* growth when using serum transferrin as a sole iron source

**Fig 7:**
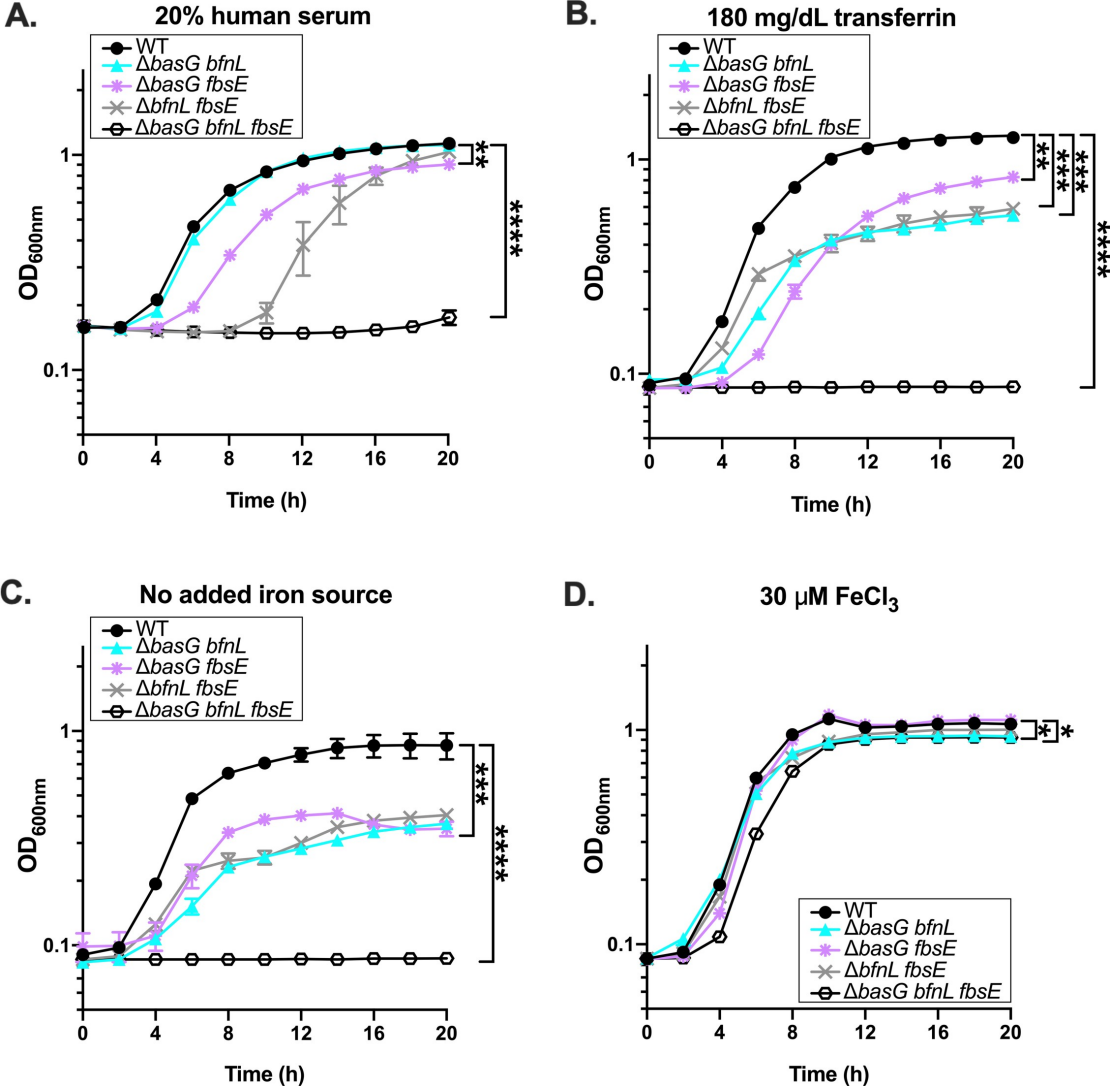
Siderophores are required for *A*. *baumannii* to utilize human serum and transferrin as iron sources to support growth. Wild-type (WT) *A*. *baumannii* and its isogenic combinatorial mutants were grown in cTMS media with 20% human serum (A), 180 mg/dL human transferrin (B), no added iron source (C), or 30 μM FeCl_3_ (D). Bacterial growth was assessed by determining OD_600nm_, at the time points indicated. Data are representative of three independent experiments, and error bars represent the standard error of the mean. Statistical analysis is given for the endpoint growth, as performed by repeated measures two-way ANOVA, where *p < 0.05, ** p < 0.01, *** p < 0.001, and **** p < 0.0001. Additional analyses of growth kinetics for these data can be found in S7 Fig.

Given that the Δ*basG* mutant alone was impaired for growth on serum and transferrin, we next sought to determine if more than just acinetobactin contributes to the acquisition of iron from these sources. To reveal the overall contribution of individual siderophores to *A*. *baumannii* growth under iron restriction, WT and the combinatorial mutant strains were grown in cTMS media with 20% human serum as the sole iron source. As previously described, growth was assessed by determining the OD_600nm_ over 20 h. Under these conditions, it was observed that all three mutants were capable of growing on serum as a sole iron source (Fig 7A), with varying degrees of impairment relative to the WT. The strains expressing baumannoferrin (Δ*basG fbsE*) or acinetobactin alone (Δ*bfnL fbsE*) exhibited a substantial growth lag relative to WT, while the strain expressing fimsbactins alone (Δ*basG bfnL*) grew comparably (Fig 7A and S7 Fig). Although it is unclear how a strain expressing fimsbactins alone is capable of growing on serum as a sole iron source, possible explanations include increased expression of fimsbactins biosynthesis (S6 Fig) or the utilization of other weak chelators such as siderophore biosynthetic intermediates or shunt products (Fig 6). Notably, the complete siderophore mutant was abolished for growth on 20% serum as a sole iron source (Fig 7A). Similar results were observed when 180 mg/dL human transferrin was provided as the iron source (Fig 7B), except that the Δ*basG bfnL* mutant grew poorly under these conditions, indicating that the fimsbactins or another chelator may scavenge residual iron from serum, but are less efficient at acquiring iron from unsaturated human transferrin. Again, all of the strains grew poorly in the absence of an iron source (Fig 7C), but growth was restored in the double mutants with the addition of exogenous iron (Fig 7D). Even with excess iron added, the siderophore-deficient strain exhibited a reproducible growth lag and reduced final biomass relative to WT (Fig 7 and S7 Fig), likely due to the absence of a high affinity means of facilitating iron uptake. Together these results highlight a high degree of functional redundancy between acinetobactin, baumannoferrins, and fimsbactins, and show that siderophore expression overall is essential to growth of *A*. *baumannii* on host iron-binding glycoproteins.

### Acinetobactin, baumannoferrin, and fimsbactins biosynthesis and transport genes are upregulated during infection

To begin dissecting the potential functional redundancy of acinetobactin, baumannoferrin, and fimsbactins *in vivo*, a murine model of systemic infection was employed to assess the expression of the siderophore biosynthesis and utilization pathways during infection using NanoString technology. NanoString is a digital, amplification-free means of quantifying targeted transcripts using color-coded molecular probes [64,65], and has been shown to reliably detect pathogen gene expression in complex samples [66,67]. In preparation for NanoString, female C57BL6/J mice were infected systemically with 2 x 10^8^ to 5 x 10^8^ colony forming units (CFUs) of WT *A*. *baumannii* by retroorbital injection. After 24 h, mice were humanely euthanized, and the organs were harvested. Following homogenization, RNA was extracted from the infected tissues, hybridized to the custom oligonucleotide probe set, and analyzed as per manufacturer instructions. As the *in vitro* comparator, RNA was similarly extracted and analyzed from bacteria grown in the same manner as used to prepare the inoculum for infection.

**Fig 8:**
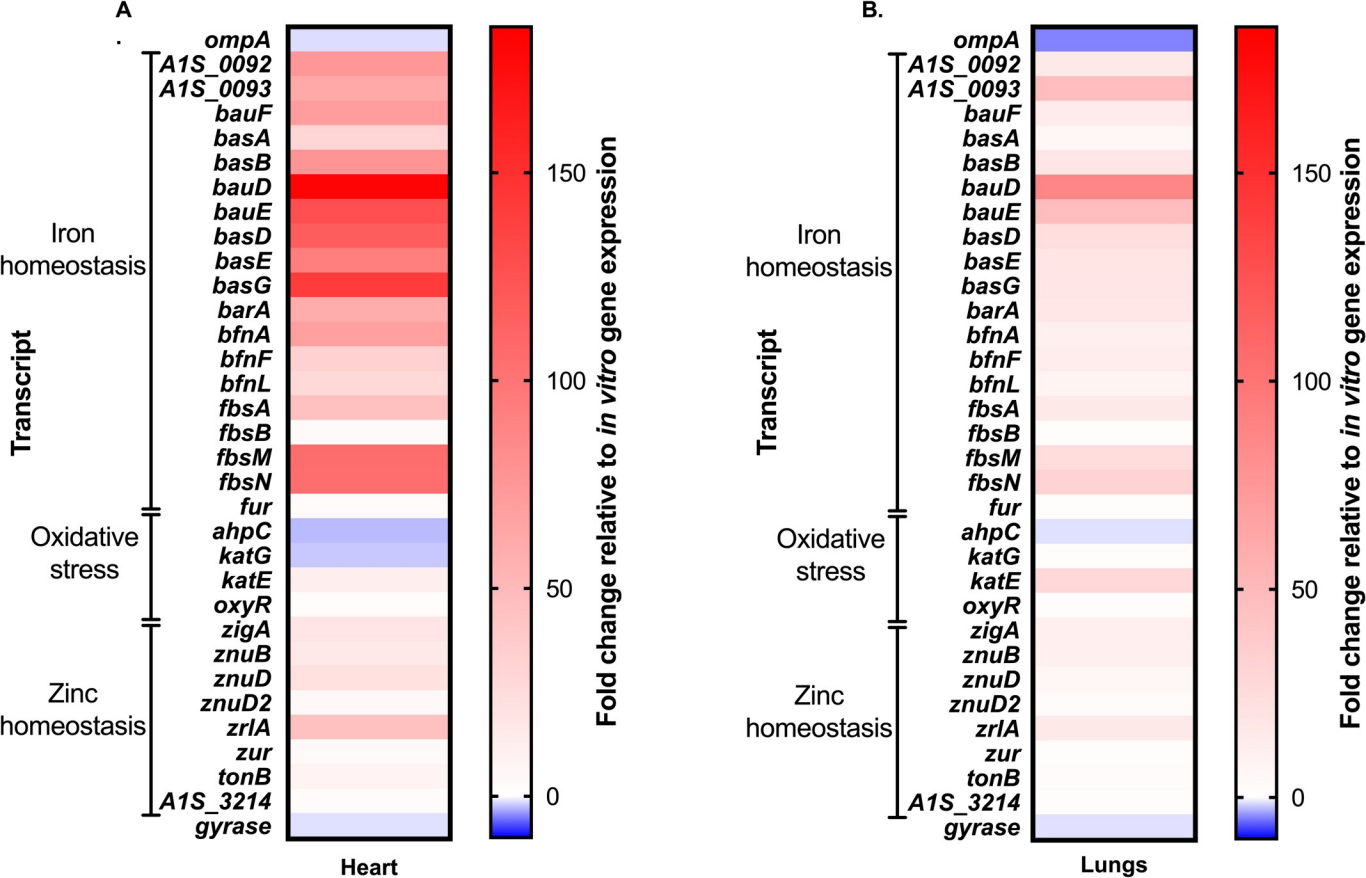
Siderophore biosynthetic and transport genes are upregulated *in vivo*. Mice were systemically infected with WT *A*. *baumannii* and sacrificed at 24 h post-infection. Organs were harvested, RNA extracted, and gene expression changes relative to growth *in vitro* were determined in the heart (A) and lungs (B) using NanoString technology. Genes are clustered by known or predicted function, as indicated.

Consistent with the *in vitro* qRT-PCR results (Fig 2A), all three siderophore loci were found to be broadly upregulated in the heart (Fig 8A) and lungs (Fig 8B) of the *A*. *baumannii* infected host, relative to bacteria grown *in vitro*. Similar results were observed in the kidneys, liver, and spleen (S8 Fig). Although the overall magnitude of gene expression varied between organs, likely due to the bioavailability of iron to the pathogen, no other major differences were observed in the expression of the three siderophore loci. These results suggest that the siderophore pathways are upregulated overall in the iron-restricted host and that the presence of functionally redundant siderophores cannot be readily explained by differential expression to facilitate growth in specific host niches.

### Acinetobactin biosynthesis is essential for the survival and proliferation of *A*. *baumannii* within the host

Since all three siderophore pathways were found to be upregulated during infection, it remained unclear as to what system, if any, is important to *A*. *baumannii* pathogenesis and whether the siderophores exhibit differential utility in specific host tissues. To elucidate the function of the individual siderophores *in vivo*, the same murine model of infection described above was employed, but instead mice were infected with WT *A*. *baumannii* or one of the single or combinatorial biosynthetic mutants. At 24 h post-infection the mice were humanely euthanized, the kidneys, hearts, livers, spleens, lungs, and blood were harvested, and the bacterial burdens of each were determined.

**Fig 9:**
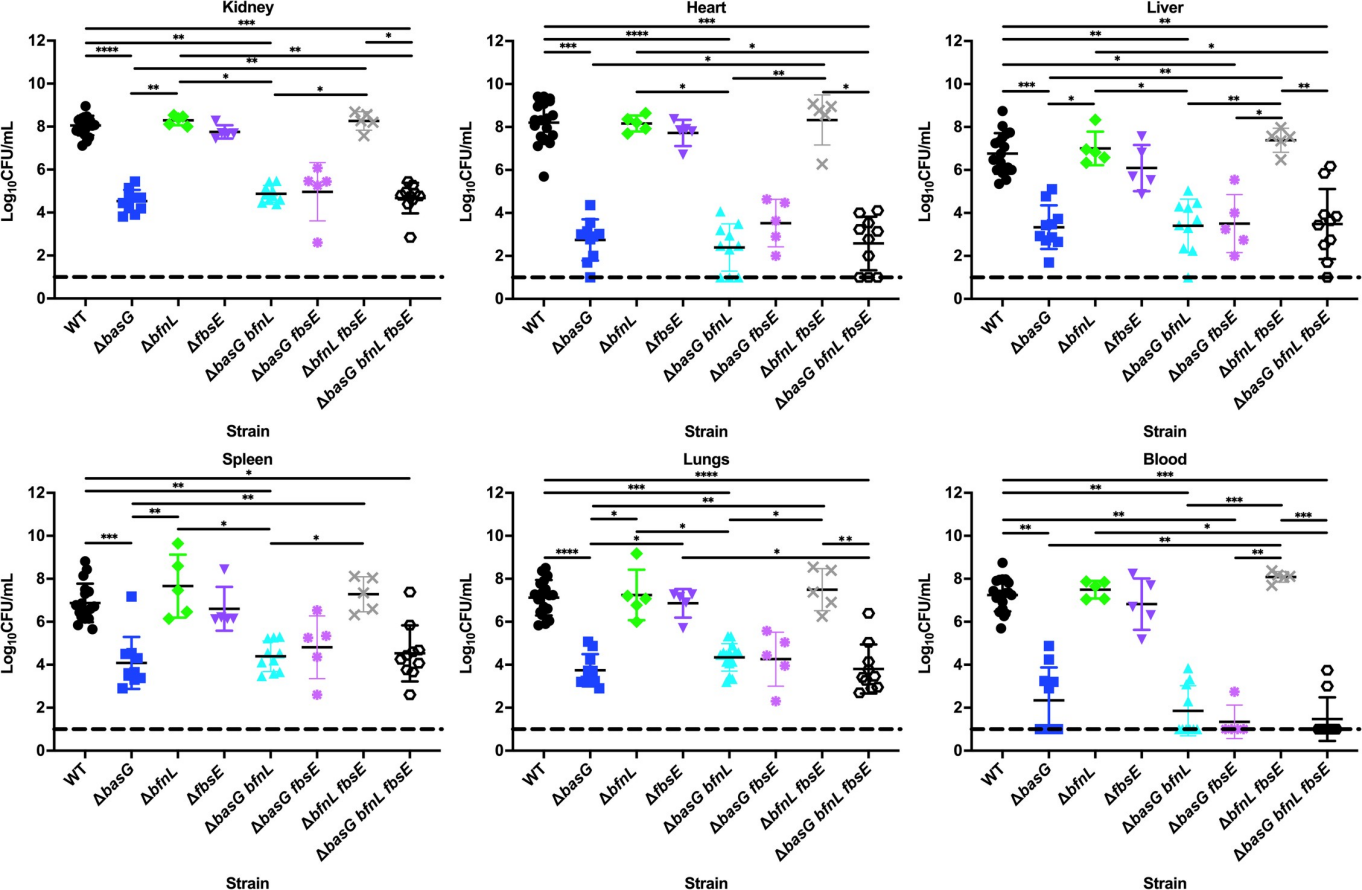
Acinetobactin biosynthetic mutant is severely attenuated for survival and proliferation within the host. Mice were systemically infected with WT *A*. *baumannii* or its isogenic siderophore biosynthetic mutants, as indicated. After 24 h, mice were sacrificed and the bacterial burdens of the kidneys, heart, liver, spleen, lungs, and blood were determined by plating for viable cell counts on lysogeny agar. Each symbol represents the *A*. *baumannii* count in the corresponding organ of one animal. Data are compiled from three independent experiments. Statistical significance was determined by Kruskal-Wallis with Dunn’s multiple comparisons test, where *p < 0.05, ** p < 0.01, *** p < 0.001, and **** p < 0.0001.

In support of the results demonstrating that acinetobactin is required for acquisition of iron from host sources such as serum transferrin and lactoferrin, the Δ*basG* mutant was found to be severely attenuated *in vivo*. Relative to WT, the acinetobactin-deficient Δ*basG* mutant had reduced bacterial burdens recovered from every organ and the blood, in the range of 2.8 to 5.0 log_10_ (Fig 9). Conversely, the Δ*bfnL* and Δ*fbsE* mutants did not exhibit a statistically significant reduction in burdens in any organ or in the blood relative to WT. Although the strain producing fimsbactins alone was capable of supporting growth of *A*. *baumannii* in serum (Fig 7A), neither this siderophore nor baumannoferrin were required for survival during *A*. *baumannii* bacteremia, as both Δ*basG bfnL* and Δ*basG fbsE* were recovered in similar numbers to the Δ*basG* mutant alone. Conversely, the strain producing acinetobactin alone (Δ*bfnL fbsE*) was not statistically different from WT. Lastly, disrupting all three siderophore biosynthetic pathways in the Δ*basG bfnL fbsE* mutant did not appreciably decrease the survival of the bacteria *in vivo* relative to Δ*basG*. Together, these results suggest that acinetobactin alone is required for the survival and pathogenesis of *A*. *baumannii* during bacteremia, whilst the function of the baumannoferrins and fimsbactins *in vivo* remains unclear.

## Discussion

The expression of multiple, structurally distinct but functionally similar siderophores is a common feature of many bacteria [29]. For bacterial pathogens, the utilization of more than one siderophore class may fulfill a number of purposes, such as avoiding siderophore piracy by other bacteria and promoting competitiveness, facilitating iron acquisition under different physiological conditions and/or iron source composition, promoting uptake of metals other than iron, and most importantly, helping to evade nutritional immunity [29,68]. Although most *A*. *baumannii* clinical isolates possess the loci for acinetobactin and baumannoferrin biosynthesis and utilization, the individual contributions of these siderophores *in vitro* and *in vivo* have not been fully elucidated. Furthermore, although the synthesis pathway and structures of the fimsbactins have been characterized [43,55,56], their function in *A*. *baumannii* growth and survival has never been interrogated using strains that are specifically inactivated for fimsbactins biosynthesis. Here we investigate the function of the individual *A*. *baumannii* siderophores using a series of single and combinatorial biosynthetic mutants. We demonstrate that all three siderophore biosynthetic pathways contribute to iron chelation *in vitro* and functional redundancy exists in their ability to facilitate iron mobilization from host sources. However, acinetobactin is essential to the efficient use of iron from host iron-binding glycoproteins, and to the survival and dissemination of *A*. *baumannii* in a murine model of bacteremia. These findings support an often stated, but not fully substantiated assertion that acinetobactin is the major, or most important, siderophore produced by *A*. *baumannii* [44].

In the context of *A*. *baumannii* bacteremia, it remains unclear why acinetobactin biosynthesis is indispensable to colonization and survival, especially with the availability of multiple alternative mechanisms for iron acquisition. Acinetobactin has been proposed to function as a “two-for-one” siderophore allowing for iron chelation over an expanded pH range, with pre-acinetobactin predominating under acidic conditions, and acinetobactin under neutral and basic conditions [38]. Thus, the critical importance of acinetobactin may be explained by its ability to bind iron under different physiological conditions. As acinetobactin biosynthesis is required for efficient utilization of transferrin and lactoferrin as sole iron sources (Fig 5 and S5 Fig, respectively), it is also possible that this siderophore has a higher affinity for iron than the baumannoferrins and fimsbactins, and is therefore better able to appropriate iron from the host. However, whilst the affinity of the baumannoferrins for iron has not been elucidated, pre-acinetobactin binds iron with a comparable affinity to fimsbactins A (log K_*Fe*_ = 27.1 ± 0.2 versus 27.4 ± 0.2) and acinetobactin binds iron with a lower affinity (log K_*Fe*_ = 26.2 ± 0.2) [38,55], suggesting that iron affinity alone does not readily explain the importance of acinetobactin. Alternatively, ferric-acinetobactin may be captured and utilized more efficiently than the other two siderophores, supported partly by the observation that the receptor protein for acinetobactin (BauA) binds a heterotrimeric acinetobactin:preacinetobactin:Fe(III) complex or a preacinetobactin_2_:Fe(III) complex with nanomolar affinity prior to uptake of the siderophore [69]. The specificity and affinity of the predicted baumannoferrin (BfnH) and fimsbactins (FbsN) receptors have not been defined, and thus further research is required to determine if efficacy in siderophore transport contributes to the utility of the siderophores.

In addition to intrinsic factors controlling the uptake of siderophores, it is also known that during infection neutrophils release siderocalin (also known as neutrophil gelatinase associated lipocalin (NGAL), 24p3, and lipocalin-2), an acute phase protein that is capable of binding and sequestering extracellular ferric catecholate and carboxymycobactin-type siderophores, thus preventing their capture by invading pathogens [70–72]. To circumvent the effects of siderocalin, some bacteria secrete structurally modified siderophores that are incompatible with the binding site of the protein, and thus are referred to as “stealth siderophores” [73]. The interactions between *A*. *baumannii* siderophores and siderocalin has not been interrogated, but it is possible their redundancy may be explained by differing susceptibilities to sequestration by the vertebrate host. Indeed, hydroxamate siderophores such as baumannoferrins A and B are unlikely to be bound by siderocalin and thus would be free to facilitate iron acquisition *in vivo*, although our findings suggest that baumannoferrin expression is dispensable to pathogenesis in a murine bacteremia model (Fig 9). Notably, the baumannoferrins possess a lipophilic decanoic side chain, which may anchor them to the cell envelope [55,74] and potentially minimize their role in the capture of iron from the extracellular milieu. Unlike the baumannoferrins, the fimsbactins are mixed ligand, bis-catecholate monohydroxamate siderophores that are also reminiscent of tris-catecholate siderophores such as enterobactin and vibriobactin, known ligands of siderocalin [55,70,75,76]. Although not experimentally validated, the fimsbactins are predicted to bind siderocalin [55], which would effectively inactivate them *in vivo* and would be consistent with our findings that disruption to fimsbactins biosynthesis does not significantly impact the outcome of infection (Fig 9). Research by the Wencewicz group has revealed that apo-fimsbactins and holo-acinetobactin compete for the same periplasmic binding protein (BauB; Fig 1D), and hence are antagonistic *in vitro* [77]. Reductive release of iron from fimsbactins is thought to be facilitated by a putative periplasmic ferric iron reductase, FbsP, allowing for accumulation of the apo-siderophore in this compartment whilst free ferrous iron is transported into the cytoplasm by FeoABC (Fig 1D) [55]. It is possible that if the fimsbactins are sequestered by siderocalin *in vivo* that this competition is alleviated, and there may be a trade-off between an *in vivo* benefit and an *in vitro* detriment to the bacteria. However, further research is required to determine what, if any, interaction exists between the fimsbactins and siderocalin. Lastly, the essentiality of acinetobactin to *A*. *baumannii* pathogenesis suggests that this siderophore is capable of evading or overwhelming siderocalin to facilitate iron acquisition by the bacteria within the host.

In assessing the role of metals on the expression of siderophore biosynthetic clusters, our results are consistent with previous findings that the acinetobactin, baumannoferrins, and fimsbactins-encoding loci are all upregulated during iron restriction [31]. Zinc starvation could also induce expression of siderophore-associated genes (Fig 2), but these transcriptional changes were 10 to 1000-fold lower than that seen with iron. It is unclear if the influence of zinc on the expression of siderophore-associated transcripts is biologically relevant, and could be explained by similarities between the Fur and Zur box consensus sequences leading to accidental crosstalk between the two regulators [20,31]. Alternatively, if Fur binds structural zinc in *A*. *baumannii* as has been reported in other bacteria [78], prolonged zinc starvation could result in dissociation of the regulator from its cognate DNA, resulting in derepression of the locus. Whilst our results strongly support that iron limitation induces the expression of acinetobactin, baumannoferrin, and fimsbactins, the role for zinc remains unknown.

Consistent with a previous study demonstrating that all three siderophore biosynthetic clusters are upregulated in the blood [79], we found each locus to be upregulated in the heart, lungs, kidneys, liver and spleen of mice during *A*. *baumannii* bacteremia (Fig 8 and S8 Fig). Notably, we did not detect niche-dependent variation in transcription of the different siderophore loci in distinct organs, although by assessing expression in whole organ homogenates, these assays may not have had the resolution required to observe these differences. Additionally, it is possible that whilst the genes for baumannoferrin and fimsbactins biosynthesis were expressed, the siderophores were not produced. Recently, high-performance matrix-assisted laser desorption/ionization imaging mass spectrometry (MALDI-IMS) was used to reveal that *Staphylococcus aureus* exhibits differential production of its siderophores, staphyloferrin A and staphyloferrin B, between abscesses within the same infected tissue [80]. Utilization of higher resolution techniques such as MALDI-IMS that incorporate spatial detection may help confirm not only the production and relative abundances of acinetobactin, baumannoferrins, and fimsbactins but may also be used to reveal niche-specific expression of the siderophores *in vivo*. Notably, in assessing total mass recovery following the *in vitro* purification of acinetobactin and fimsbactins, Bohac *et al* observed greater mass production of the former, whilst the baumannoferrins were not detected using the methods employed [55]. The aforementioned results suggest that there may indeed be differences in overall siderophore abundances that may help to explain why acinetobactin is critically important to infection. Whilst the use of mass spectrometry to identify and quantify the production of each of the siderophores was outside the scope of this study, use of these technologies represent powerful tools going forward.

Together, the conservation of the acinetobactin locus amongst clinical isolates of *A*. *baumannii* and essentiality to survival of the pathogen *A*. *baumannii in vivo* [31,46,51], even when other siderophores are expressed, leads us to propose that developing novel therapeutics that specifically target acinetobactin biosynthesis or transport may represent a viable strategy to combat this extensively drug-resistant pathogen. Several approaches exist for targeting iron homeostasis pathways in bacterial pathogens, but two main methods include developing vaccines against iron-regulated surface-exposed antigens and coopting iron acquisition systems to deliver a toxic payload to the bacterium [22]. Antibodies raised against iron-regulated TonB-dependent transporters (TBDTs) often function both to promote osponophagocytosis and to inhibit iron uptake [81], and indeed the receptor proteins for both acinetobactin and baumannoferrins, BauA and BfnH, respectively, have shown promise as antigens for vaccine development against *A*. *baumannii* [82–84]. Although the expression of *bfnH* was not specifically assessed by NanoString in this study, both *bauA* and *fbsN* are upregulated during infection, with the latter possessing some of the highest transcript abundances in most organs (Fig 8 and S8 Fig), again providing support for the use of TBDTs as vaccine antigens. A potential limitation to vaccination against *A*. *baumannii*, is that patients afflicted by the disease are often critically ill and immunocompromised and thus unable to mount a sufficient antibody response. Passive immunization, using monoclonal antibodies, therefore represents a potential option for protecting patients in high risk environments, such as intensive care units.

An alternative strategy for exploiting iron acquisition systems to the detriment of bacterial pathogens is to covalently link either a natural or synthetic siderophore to an antibiotic and utilize the siderophore uptake systems of the bacteria to help internalize this compound, known as a sideromycin. This “Trojan horse” approach to treating bacterial infections recently gained traction with the development of cefiderocol (Fetroja), a catechol-cephalosporin conjugate that recently became the first sideromycin-type antibiotic approved by the United States Food and Drug Administration (FDA) [85]. Indicated for the treatment of complicated urinary tract infections with few or no other treatment options, cefiderocol is active against Gram-negative, multidrug-resistant pathogens such as *A*. *baumannii* [86,87]. As with other sideromycins, cefiderocol is actively transported into the cell by coopting a siderophore-TBDT [88], and its minimum inhibitory concentration decreases under iron restriction [86]. Additionally, fimsbactins A and fimsbactins-like analogues conjugated with daptomycin or cephalosporins are highly effective and selective in killing *A*. *baumannii in vitro*, showing that antibiotics typically reserved for Gram-positives may be repurposed by using a siderophore delivery mechanism to gain access to the periplasm of Gram-negative bacteria [89,90]. These findings highlight that sideromycins can be effective as antibiotics against *A*. *baumannii*. Importantly, the essential features required for iron coordination by, and cellular uptake of acinetobactin have been identified, along with a promising sites for the conjugation of an antibiotic [38,44,91]. Interestingly, in assessing the development of resistance to another experimental sideromycin (BAL30072), *A*. *baumannii* developed mutations in genes encoding the TonB machinery required to energize siderophore uptake systems, *tonB3* and *exbD3* [33,88]. Although these mutants had decreased sensitivity to the sideromycin, the *tonB3* and *exbD3* mutants exhibited decreased growth under iron restriction, suggesting that should these mutations evolve in the iron-restricted host, the bacteria may still be compromised for survival [88]. Indeed, Runci *et al*. have found that *tonB3* is required for maximal virulence of *A*. *baumannii* ATCC 19606 in insect and murine models of infection [33].

The results of this study have revealed that acinetobactin is essential for iron acquisition *in vivo*, whereas other siderophores, the baumannoferrins and fimsbactins, are dispensable. Moreover, we have demonstrated that the genes for acinetobactin biosynthesis and transport are broadly upregulated in a systemic murine infection model. Collectively these data, coupled with the observation that the acinetobactin locus is highly conserved amongst clinical isolates, presents the targeting of acinetobactin biosynthesis or transport as an attractive candidate for the development of novel therapeutics to combat multidrug-resistant *A*. *baumannii*.

## Materials and methods

### Bacterial strains and growth conditions

All bacterial strains and plasmids employed in this study can be found in Table 1. Unless otherwise indicated, experiments were performed with *A*. *baumannii* strain ATCC 17978 and its isogenic mutants. For routine cultivation and genetic manipulation, *A*. *baumannii* was cultured in lysogeny broth (LB) or on LB with 1.5% w/v agar (LBA). Antibiotics, when required for selection of recombinants or maintenance of plasmids in *A*. *baumannii*, were supplied at the following concentrations: ampicillin, 500 μg/mL; carbenicillin, 75 μg/mL; kanamycin, 15 μg/mL; sulfamethoxazole, 100 μg/mL; as indicated. For selection and maintenance of plasmids in *E*. *coli*, 100 μg/mL of ampicillin was used. Plasmid gene expression was induced using 1–2 mM isopropyl β-d-1-thiogalactopyranoside (IPTG), as detailed in the text.

**Table 1:** Strains and plasmids employed in this study.

Strain	Description	Reference
*A*. *baumannii* ATCC 17978	WT *A*. *baumannii* fatal meningitis isolate from 1951	American Type Culture Collection (ATCC) strain 5377 [54]
WT/pWH1266	WT *A*. *baumannii* ATCC 17978 with empty pWH1266 plasmid	This study
*A*. *baumannii* AB5075-UW	Multidrug-resistant WT *A*. *baumannii* AB5075 isolate from a wound infection	[92,93]
Δ*basG*	Acinetobactin biosynthetic mutant where *basG* (A1S_2379) was replaced by a kanamycin resistance determinant, and then the cassette was excised, to leave a markerless mutant	This study
*basG*102::Tn26	Transposon mutant of *basG* in *A*. *baumannii* AB5075-UW from three-allele mutant library; identifier tnab1_kr121128p06q102	[92]
*basG*142::Tn26	Transposon mutant of *basG* in *A*. *baumannii* AB5075-UW from three-allele mutant library; identifier tnab1_kr130909p02q142	[92]
Δ*basG*/pWH1266::*basG*	*basG* mutant complemented with pWH1266 expressing *basG* from its native promoter	This study
Δ*bfnL*	Baumannoferrin biosynthetic mutant where *bfnL* (A1S_1657) was replaced by a kanamycin resistance determinant, and then the cassette was excised, to leave a markerless mutant	This study
Δ*fbsE*	Fimsbactins biosynthetic mutant where *fbsE* (A1S_2578) was replaced by a kanamycin resistance determinant; Kan^R^	This study
Δ*basG bfnL*	Acinetobactin and baumannoferrin biosynthetic mutant. Proficient in fimsbactins biosynthesis; Kan^R^	This study
Δ*basG fbsE*	Acinetobactin and fimsbactins biosynthetic mutant. Proficient in baumannoferrin biosynthesis; Kan^R^	This study
Δ*bfnL fbsE*	Baumannoferrin and fimsbactins biosynthetic mutant. Proficient in acinetobactin biosynthesis; Kan^R^	This study
Δ*basG bfnL fbsE*	Complete siderophore biosynthetic mutant with disruptions in acinetobactin, baumannoferrin, and fimsbactins production; Kan^R^	This study
Δz*ur*	Zur mutant; Kan^R^	[58]
**Plasmid**		
pKD4	Template for amplification of the FRT-flanked kanamycin resistance determinant; Kan^R^, Amp^R^	[94]
pAT02	*A*. *baumannii* recombinase expressing plasmid; Carb^R^/Amp^R^	[61]
pAT03	Expresses an IPTG-inducible flipase to remove the kanamycin resistance determinant; Carb^R^/Amp^R^	[61]
pWH1266	*A*. *baumannii*/*E*.*coli* shuttle and complementation vector; Carb^R^/Amp^R^	[95]
pWH1266::*basG*	pWH1266 expressing *basG* from its native promoter; Carb^R^/Amp^R^	This study

For bacterial growth under metal-restriction, media were prepared in sterile polypropylene vessels or in glassware that was washed overnight with 0.1 M HCl and then subsequently rinsed extensively with ultrapure water to remove contaminating iron. Media and all components were prepared using water purified with a Nanopure Diamond filtration system (Thermo Scientific). When required, base media and additives were Chelex-treated using 5% w/v Chelex-100 resin (Sigma-Aldrich) stirring overnight at 4°C. Tris Minimal Succinate (TMS) media were employed in zinc and iron-restriction studies, prepared as previously described [96]. For growth curves, TMS was treated with Chelex-100 (cTMS) to remove residual iron.

### RNA extraction and quantitative reverse transcription PCR

To assess *in vitro* gene expression, WT *A*. *baumannii* or its isogenic mutants were grown overnight in TMS media prior to subculturing 1:50 in 10 mL cTMS media with or without the addition of 30 μM ZnCl_2_ or FeCl_3_. Bacteria were grown until exponential (4 h) or stationary phase (12 h), pelleted by centrifugation, and siderophore activity was confirmed in metal-deplete culture supernatants (see “Determination of siderophore production by Chrome Azurol S assay”). Bacterial pellets were stored in a 1:1 solution of acetone and ethanol at -80°C until RNA extraction. Prior to extraction, the acetate:ethanol was removed by aspiration following centrifugation for 15 min at maximum speed. The bacterial pellets were resuspended in 750 μL of LETS buffer (0.1 M LiCl, 10 mM EDTA, 10 mM Tris HCl (pH 7.4), 1% sodium dodecyl sulphate) and lysed in Lysing Matrix B tubes using a FastPrep-24 bead beater (MP Biomedicals) with two 45 s pulses at 6 M/s separated by a 5-min rest. Following incubation at 55°C for 5 min, the samples were centrifuged at 21,130 *x g* for 10 min and then transferred to new 2 mL RNAase-free tubes. RNA was solubilized by mixing lysates with 1 mL TRI reagent (Sigma-Aldrich) and incubated at room temperature for 5 min. To each sample, 0.2 mL of chloroform was added and mixed by rapid inversion for 15 s. Following incubation at room temperature for 2–3 min, the samples were centrifuged at 21,130 *x g* for 10 min, and the upper aqueous phase was transferred to a new tube and mixed with 1 mL of isopropanol. RNA was precipitated at room temperature for 10 min, pelleted by centrifugation, washed with 200 μL of 70% ethanol, and dried at room temperature for 1 min. The RNA pellet was resuspended in 100 μL of nuclease-free water and treated with RNA Qualified (RQ1) RNase-Free DNase for 2 h at 37°C (Promega). RNA was then purified further using an RNeasy miniprep kit, as per instructions from the manufacturer (Qiagen). RNA quality and quantity were assessed using a NanoDrop 8000 Spectrophotometer (Fisher Scientific).

cDNA was synthesized from 1 μg RNA using random primers and M-MLV reverse transcriptase, using instructions from the supplier (Promega). The cDNA was diluted 1:50 and used in quantitative reverse transcription PCR (qRT-PCR) using the primer pairs denoted in Table 2 and iQ SYBR Green Supermix, as directed (Bio-Rad). qRT-PCR was performed using a two-step melt curve program on a CFX96 Touch Real-Time PCR detection system (Bio-Rad). Target gene expression was normalized to the expression of *rpoB* and presented as the fold change (2^-ΔΔCT^) relative to expression under metal-replete conditions, or to WT when gene expression was assessed in the Δ*zur* or Δ*basG bfnL* mutants.

**Table 2:** Primers employed in this study.

Designation	^a^^,^^b^Sequence	Function
*basE*-F RT	CGCGCATTTTAACGGTAGGG	*basE* qRT-PCR
*basE*-R RT	CCCTTGCCCAAGCCTTTTTC	*basE* qRT-PCR
*basD*-F RT	GGCAACGCAACTTTAGGTGG	*basD* qRT-PCR
*basD*-R RT	AGCCATGATGTTTGCGATGC	*basD* qRT-PCR
*bauD*-F RT	GTATCGGGTTGGCGGTATGT	*bauD* qRT-PCR
*bauD*-R RT	ATCATCTTGCTCAGCGTGCT	*bauD* qRT-PCR
*basB*-F RT	CAAAATGCCAAAGGTCGCCA	*basB* qRT-PCR
*basB*-R RT	GCTTTCCAGTTTGGGGCTTG	*basB* qRT-PCR
*basA-*F RT	TATGGATTCTCCGCCATCGC	*basA* qRT-PCR
*basA*-R RT	AGCCGGACGTCTGTTGATTT	*basA* qRT-PCR
*bauF*-F RT	ATTGTCTCGATCCAGACGCC	*bauF* qRT-PCR
*bauF*-R RT	TTGCCAAATTGGGAGCTTGC	*bauF* qRT-PCR
*bfnA*-F RT	TCGGTTTACGTGGATGGCAA	*bfnA* qRT-PCR
*bfnA*-R RT	TCGCCACGATGACACTCTTT	*bfnA* qRT-PCR
*bfnF*-F RT	AGTCAAAAGCTGCCGAAAGT	*bfnF* qRT-PCR
*bfnF*-R RT	ACGGCATAAGGTCTGCTCAG	*bfnF* qRT-PCR
*bfnL*-F RT	TACACCGCTGGATGCATGAG	*bfnL* qRT-PCR
*bfnL*-R RT	AATTTCGGCATACCCCACGT	*bfnL* qRT-PCR
*fbsA*-F RT	TCACCTCTCCCCAACCATCA	*fbsA* qRT-PCR
*fbsA*-R RT	GGCGACTCAGCACTCATCTT	*fbsA* qRT-PCR
*fbsB*-F RT	AGCCTTGGCATTGTCTCTCC	*fbsB* qRT-PCR
*fbsB*-R RT	ATCCATCCACCCGACCAAAC	*fbsB* qRT-PCR
*fbsM*-F RT	GTGTTCCCTCTGCAGGTAGC	*fbsM* qRT-PCR
*fbsM*-R RT	CTTTCGTCCATGACCAGGGT	*fbsM* qRT-PCR
*fbsN*-F RT	ACCGTTATTTGGGTTCGGCT	*fbsN* qRT-PCR
*fbsN*-R RT	AATCGCACCACGTGTTTTGG	*fbsN* qRT-PCR
*basG*-F RT	AGCGCAAATCGGAATCATGC	*basG* qRT-PCR
*basG*-R RT	TGGCCAGACACACAAATCGA	*basG* qRT-PCR
*fur-*F RT	TGCGCAAAGCTGGACTTAAA	*fur* qRT-PCR
*fur-*R RT	TCGCAAGTCCGACATCTTCC	*fur* qRT-PCR
*zur-*F RT	GTCACCCACGTGAAGGTCAT	*zur* qRT-PCR
*zur-*R RT	CTGTGTTGTGCTGCGAAATC	*zur* qRT-PCR
*zigA*-F RT	GATCAGGCTCAGCAAGACCAG	*zigA* qRT-PCR
*zigA*-R RT	GTGCTTGGACAGCTTCATCA	*zigA* qRT-PCR
*rpoB*-F RT	ATGCCGCCTGAAAAAGTAAC	*rpoB* qRT-PCR (housekeeping)
*rpoB*-R RT	TCCGCACGTAAAGTAGGAAC	*rpoB* qRT-PCR (housekeeping)
*gyrA*-F RT	GACGACGGTACCGGTTTACA	*gyrA* qRT-PCR (housekeeping)
*gyrA*-R RT	ACCGCGGCCATTATCTGAAA	*gyrA* qRT-PCR (housekeeping)
*basG*-F	CAGGGTAGAGGGTTGCCATCATAAGGCATACACAAATCCTGGTCGCTAAATTTATACATAAAATTTATATCTTTGATAGCGACTCCTTAGACGACTTGTAATCGTCATATTAACGGAGCAAAAAGTGTAGGCTGGAGCTGCTTC	Recombineering to replace *basG* with Km^R^
*basG*-R	AGTATTTTTTTAAAAACTTTAATTGCATCAAAAACTTCCTAACCACCCTATCAAAATATTTTTAGATCATTTTCTAGACTGTAAAAATTAATATGGATTTGTCTAAATCATCTTGGTTGATATGACATATGAATATC CTCCTTAG	Recombineering to replace *basG* with Km^R^
Full *basG*-F	GTATCTTCACGTTGCGGTCAGGTC	Checking recombineering of *basG*
Full *basG*-R	AATGCTGGCTTGAGTGCAGGT	Checking recombineering of *basG*
*bfnL*-F	GGGGAAAGCTGAATTTTTTGTCCATGATTTATGCAATAAAAAAAATAGTAAAAATAAGCTTGCATCTTAAATAAAAATGATTATCATTATCAATTATAGGTTATGTCATAGCAAGGACCGTTATAGTGTAGGCTGGAGCTGCTTC	Recombineering to replace *bfnL* with Km^R^
*bfnL*-R	AACTCCTGCCCAATGAGTTCTTGAGATGCCTGTTGCAATGTTTGAAACCGCAACCGTTCAGTATCAAACATTGTCCCATCCATATCGAAGATAGCTCCATGAACAGGTTTTCCATGAAAAATAAGCATATGAATATCCTCCTTAG	Recombineering to replace *bfnL* with Km^R^
Full *bfnL*-F	TGTCGATCTGGCGCTCATAC	Checking recombineering of *bfnL*
Full *bfnL*-R	GGTTCCTGTATCTCTGGCGT	Checking recombineering of *bfnL*
*fbsE*-F	ATGCAATTACAACAAGAAATTACCGCAGACATTCACCATGTTCTGCAAATTGATAGCATTCAAATTCAACCTGAAGATAATTTGATTGAACATGGGCTGCATTCATTGGCGATCATGCAGTTAGTGTGTAGGCTGGAGCTGCTTC	Recombineering to replace *fbsE* with Km^R^
*fbsE*-R	ATCACTTGCATCAATTGCATTTCATCGATGGTGGTACGTAATGCAGGATATAATGCAATCAATTGAGTCAAACGCTGAGTCAGTGTTTCAACATCCATTCGCCCATGAAATTCTTGAAAGTCATGCATATGAATATCCTCCTTAG	Recombineering to replace *fbsE* with Km^R^
Full *fbsE*-F	AACTGTGGGGTTGGAACTCG	Checking recombineering of *fbsE*
Full *fbsE*-R	TGAATGGTCGCTTCCATGCT	Checking recombineering of *fbsE*
*basG* Gibson-F	gcgaccacacccgtcctgtgTTGGACTCATTACGAATTATG	Complementation of *basG*
*basG* Gibson-R	aaggctctcaagggcatcggCTAAAAGCCAACTGTACG	Complementation of *basG*

^a^Underlined areas denote regions of homology with the kanamycin resistance determinant

^b^Lower case letters denote regions of homology with pWH1266

### Generation of *A*. *baumannii* siderophore biosynthetic mutants

Oligonucleotides used in generating, confirming, or complementing the siderophore biosynthetic mutants detailed below can be found in Table 2. All mutants were generated using recombineering, as previously described [61]. In brief, the FRT-flanked kanamycin resistance determinant of pKD4 [94] was amplified using primers bearing 120 bp of homology to the region flanking the gene of interest (*basG*, *bfnL*, or *fbsE*, using primers *basG-*F/R, *bfnL*-F/R, and *fbsE-*F/R, respectively). The PCR product was purified and subsequently concentrated to ~1–2 μg/μL. Approximately 5 μg of linear recombineering product was introduced to 100 μL (~10^8^ colony forming units (CFU)) of competent *A*. *baumannii* containing the recombinase-expressing plasmid pAT02 [61] by electroporation at 1800 V. Cells were recovered immediately in pre-warmed LB, and recombinase expression was induced through the addition of 2 mM of IPTG to the media. After 4 h of growth at 37°C, the transformants were plated to LBA with kanamycin. The putative Δ*basG*::km, Δ*bfnL*::km or Δ*fbsE*::km colonies that arose on the transformation plates were screened by PCR using primer pairs Full *basG-*F/R, Full *bfnL-*F/R, or Full *fbsE-*F/R, respectively, each of which flank the site of recombination. Colonies were screened for the loss of pAT02 through patching to LBA supplemented with carbenicillin, and maintenance of the native plasmid pAB3 was confirmed through resistance to sulfamethoxazole [97]. The *basG* mutants obtained from the *A*. *baumannii* AB5075-UW three-allele mutant library [92] were confirmed through PCR using primer pairs Full *basG*-F/R.

In order to generate combinatorial mutants, it was necessary to excise the kanamycin cassette from the single siderophore biosynthetic mutants to yield unmarked mutations. The strains Δ*basG*::km and Δ*bfnL*::km were transformed with the Flp recombinase plasmid, pAT03 [61]. Expression of the flippase was induced by growth of Δ*basG*::km/pAT03 and Δ*bfnL*::km/pAT03 in LB with 1 mM IPTG for 4 h. The cells were harvested by centrifugation, plated to LBA containing 1 mM IPTG, and incubated overnight at 37°C. Biomass was scraped from the induction plates and streaked for isolated colonies on LBA without antibiotics. Colonies were screened for excision of the kanamycin cassette and simultaneous loss of the pAT03 plasmid through identifying isolates that were both kanamycin and carbenicillin sensitive. Excision of the kanamycin cassette was confirmed through colony PCR using primer pairs Full *basG-*F/R or Full *bfnL-*F/R.

Upon generation of the markerless strains, combinatorial mutants were generated in the Δ*basG* and Δ*bfnL* backgrounds. The procedure was performed as described above, except that the linear DNA fragment bearing homology to *fbsE* was introduced to both Δ*basG*/pAT02 and Δ*bfnL*/pAT02, and the linear DNA fragment bearing homology to *bfnL* was also introduced to Δ*basG*/pAT02. Screening and confirmation of the Δ*basG bfnL*::km, Δ*basG fbsE*::km, and Δ*bfnL fbsE*::km mutants was performed as detailed above. A complete siderophore biosynthetic mutant was generated by excising the kanamycin cassette from Δ*basG bfnL*::km, and subsequently disrupting *fbsE*. All mutations were confirmed through PCR and maintenance of pAB3 was assured through resistance to sulfamethoxazole.

### Complementation of *basG*

Complementation of *basG* was performed *in trans* using the vector pWH1266 [95]. The *basG* gene and its native promoter were amplified using the primers *basG* Gibson-F/R (Table 2). pWH1266 was digested with BamHI and SalI and the PCR product and plasmid were joined using NEB Builder HiFi Assembly (New England BioLabs) at 50°C for 1 h. The resulting assembly was transformed into chemically competent DH5*α* by heat shock, and successful transformants were selected for on LBA with ampicillin following overnight growth at 37°C. The pWH1266::*basG* construct was confirmed by PCR and sequencing prior to introduction of both this vector, as well as the native empty vector, to Δ*basG* by electroporation. Successful transformants were selected on LBA with carbenicillin.

### Determination of siderophore production by Chrome Azurol S assay

To assess for overall siderophore production, Chrome Azurol S (CAS) assays were performed. In preparation for the assays, bacteria were grown in biological triplicate overnight in TMS media, pelleted by centrifugation at 1073 *x g* and washed three times with 1X phosphate buffered saline (PBS). The OD_600_ of each resuspension was adjusted to 1.0 and used to inoculate 10 mL of cTMS media to an OD_600_ of 0.005 in 50 mL conical tubes. The cultures were grown for 12 h at 37°C with shaking at 180 rpm. Following growth, the OD_600nm_ of each culture was determined and bacteria were pelleted by centrifugation. The supernatants were removed and sterilized using a 0.2 μm filter prior to use in the CAS assays.

CAS reagent was prepared, and the assays performed, essentially as described previously [59,60]. In brief, CAS is an iron-binding dye complex that detects mobilization of iron from the dye to a chelator through a colorimetric change from blue (negative reaction) to pink/orange (positive reaction) and/or through a decrease in absorbance at 630 nm (A_630_). Prepared CAS reagent was mixed 1:1 with the filtered culture supernatants and incubated in the dark at room temperature for 30 min. The A_630_ was determined, each value was normalized to the final OD_600nm_ of the corresponding culture, and siderophore activity was expressed as a percentage of WT *A*. *baumannii*.

### Assessing the ability of *A*. *baumannii* to utilize host iron sources under iron-restricted growth

To assess growth of *A*. *baumannii* WT and the siderophore biosynthetic mutants under iron-restriction, single-isolated colonies of bacteria grown on LBA were used to inoculate 3 mL of TMS media and incubated overnight at 37°C with shaking at 180 rpm. The overnight cultures were pelleted by centrifugation at 604 *x g* for 15 min and washed once with sterile 1x PBS. The pellets were resuspended in PBS and subcultured 1:100 in cTMS media. Cultures were grown to an OD_600nm_ of ~1, pelleted by centrifugation, washed three times with sterile 1 x PBS and resuspended to an OD_600nm_ of 0.1. Growth curves were set-up in cTMS in 96-well plates using 20% v/v heat inactivated and filter sterilized human serum (Sigma-Aldrich; H4522), 180 mg/dL partially saturated human transferrin (Sigma-Aldrich; T3309), or 15 mg/mL lactoferrin from human milk (Sigma-Aldrich; L0520). Concentrations were selected to be physiologically relevant and to support growth of WT *A*. *baumannii* in the absence of another iron source. For zinc and iron-replete conditions, 30 μM of ZnCl_2_ or FeCl_3_ were added, respectively. Carbenicillin was added when required to maintain pWH1266 or its derivatives. Plates were inoculated using 5 μL of the prepared cells in 200 μL of media and grown in an Epoch 2 microplate reader (BioTek) at 37°C with medium amplitude linear shaking. Cell density was assessed by OD_600nm_ every 30 min, but for graphical clarity only data collected at 4 h intervals is shown. Estimations of the asymptote of the maximal OD_600nm_ (A)_,_ maximum specific growth rate (μ_m_) and lag time (λ) were modeled using single Gompertz growth curve fitting and open source code available at https://scott-h-saunders.shinyapps.io/gompertz_fitting_0v2/ [98–100].

### Murine model of *A*. *baumannii* bacteremia

All infection experiments were approved by the Vanderbilt University Institutional Animal Care and Use Committee and are in compliance with guidelines set by the Animal Welfare Act, the National Institutes of Health, and the American Veterinary Medical Association. Six-to-eight- week old female C57BL/6J mice (Jackson Laboratories) were injected retro-orbitally with 100 μL of bacterial cell suspensions containing ~2 x 10^8^ to 5 x 10^8^ colony forming units (CFU) of WT *A*. *baumannii* or its isogenic mutants in PBS. In preparation for infection, overnight cultures of bacteria were subcultured 1:100 in fresh LB and grown to mid-to-late log phase (OD_600_ = 2.0–2.5) at 37°C with shaking. The bacteria were pelleted by centrifugation, washed twice with PBS, and normalized to an OD_6oonm_ of 0.35 at a 1:20 dilution. Following injection, the mice were humanely euthanized at 24 h, and the kidneys, heart, liver, spleen, lungs, and blood of each mouse were harvested. The organs were homogenized in PBS using a Bullet Blender (Next Advance), and all samples were serially diluted in PBS prior to plating on LBA to determine the bacterial burdens.

### RNA extraction and *in vivo* gene expression analysis by NanoString

For assessing *in vivo* gene expression, mice were infected as detailed above. At 24 h, mice were humanely euthanized, and the harvested organs were placed in RNAase-free navy bullet blender tubes (Next Advance) containing 600 μL of RLT buffer (Qiagen) with 1% (v/v) β-mercaptoethanol. Following homogenization in the bullet blender for 2 x 5 min (speed 8 for livers, speed 10 for all other organs; Next Advance), the homogenate was transferred to a fresh bullet blender tube containing 600 μL of phenol:chloroform:isoamyl alcohol (25:24:1, Fisher Scientific). After an additional 5 min homogenization cycle, the samples were centrifuged at 21,139 *x g* for 10 min. The upper aqueous phase was transferred to a 2-mL nuclease-free tube containing 600 μL of 70% ethanol and inverted until a visible mass of RNA formed. The extracted RNA was subsequently purified using an RNeasy kit (Qiagen), as per manufacturer’s instructions. As an *in vitro* comparator, WT *A*. *baumannii* was grown in LB using the same methodology used to prepare for infection (see section “Murine model of *A*. *baumannii* bacteremia”). RNA was extracted from *in vitro* grown bacteria using the method described in “RNA extraction and quantitative reverse transcription PCR”. RNA quality and concentration were determined using a NanoDrop 8000 Spectrophotometer (Fisher Scientific).

In preparation for NanoString, 100 ng of RNA in 5 μL of nuclease-free water was aliquoted to 12-well tube strips and mixed with 8 μL of the Reporter Probe Set in hybridization buffer (NanoString). To each well, 2 μL of the Capture Probe Set (NanoString) were added and the samples were hybridized at 65°C in a preheated thermocycler for 18 h. Following hybridization, samples were processed by Vanderbilt Technologies for Advanced Genomics (VANTAGE) on an nCounter FLEX analysis system (NanoString). Data were analyzed using nSolver Analysis software (NanoString), where *A*. *baumannii* gene expression was normalized to the expression of housekeeping genes *rpsA*. Using negative control probes included by the manufacturer in the Reporter Probe Set and designed not to interact with biological samples (NanoString), the minimum threshold for gene expression was determined and all target genes included in the analysis exhibited expression above background. Expression changes are given as the fold change of *in vivo A*. *baumannii* gene expression versus gene expression in *in vitro* grown bacteria.

## Supporting information

S1 Table Genes involved in acinetobactin biosynthesis and utilization in *A*. *baumannii* ATCC 17978. (DOCX)

S2 Table Genes involved in baumannoferrin biosynthesis and utilization in *A*. *baumannii* ATCC 17978. (DOCX)

S3 Table Genes involved in fimsbactins biosynthesis and utilization in *A*. *baumannii* ATCC 17978. (DOCX)

S1 Fig Zinc may play a minor role in the regulation of siderophore-associated genes in *A*. *baumannii* but does not appear to be involved in the direct transcriptional regulation of *fur*. WT *A*. *baumannii* and its isogenic Δ*zur* mutant were grown in metal-restricted media for 12 h. RNA was extracted and transcriptional changes in the expression of siderophore-associated genes and *fur* were assessed by qRT-PCR and normalized to the expression of *rpoB*. Expression of a known *zur*-regulated gene, *zigA*, was assessed as a positive control, whereas *zur* was run as a negative control. * p < 0.05, *** p < 0.001, and **** p < 0.0001 as determined by Student’s *t* test relative to a hypothetical value of 1. Data are representative of two experiments performed in biological quadruplicate.
(TIFF)

S2 Fig Acinetobactin biosynthetic mutants exhibit decreased maximal growth and growth rates, as well as an increased lag time relative to WT *A*. *baumannii* ATCC 179789 when grown under iron restriction. Growth kinetics of WT *A*. *baumannii* ATCC 17978 and its isogenic acinetobactin (*ΔbasG*), baumannoferrin (Δ*bfnL)* and fimsbactins (Δ*fbsE*) biosynthetic mutants were analyzed from the data presented in Fig 5. Estimates of the maximal OD_600_ (asymptote (A) A-D), maximum specific growth rate (μ_m_, E-H) and lag time (λ, I-L) are given where *p < 0.05, ** p < 0.01, *** p < 0.001, and **** p < 0.0001.
(TIFF)

S3 Fig Expression of *basG in trans* restores growth of *A*. *baumannii* 17978 on human serum. WT *A*. *baumannii* ATCC 17978 with empty vector (WT/pWH1266), the acinetobactin-deficient mutant with empty vector (Δ*basG*/pWH1266), and a mutant complemented with *basG* expressed from pWH1266 (Δ*basG*/pWH1266::*basG*) were grown in cTMS media with human serum added at the concentrations indicated. Bacterial growth was assessed by determining the optical density at 600 nm (OD_600nm_) at the timepoints indicated. Data are the average of technical triplicates and represent the results of two independent experiments.
(TIFF)

S4 Fig Acinetobactin biosynthetic mutants in *A*. *baumannii* AB5075-UW are deficient for growth in human serum. WT *A*. *baumannii* AB5075-UW and two unique *basG* transposon mutants were grown in cTMS media with human serum added at the concentration indicated. Bacterial growth was assessed by determining the OD_600nm_ at the timepoints indicated. Data are the average of technical triplicates and represent the results of two independent experiments. Where error bars are not visible, they are shorter than the height of the symbol.
(TIFF)

S5 Fig Acinetobactin biosynthetic mutants are impaired for growth on lactoferrin as a sole iron source. Wild-type (WT) *A*. *baumannii* and its isogenic acinetobactin (Δ*basG*), baumannoferrin (Δ*bfnL)* and fimsbactins (Δ*fbsE*) biosynthetic mutants were grown in cTMS media with lactoferrin, no added iron source, or 30 μM FeCl_3_, as indicated. Bacterial growth was assessed by determining the OD_600nm_, at the time points indicated. Data are representative of two independent experiments, and error bars represent the standard error of the mean. Where error bars are not visible, they are shorter than the height of the symbol.
(TIFF)

S6 Fig Fimsbactins biosynthetic, regulatory, and transport genes are upregulated in a Δ*basG bfnL* mutant. WT *A*. *baumannii* and its isogenic Δ*basG bfnL* mutant were grown in metal-restricted media for 12 h. RNA was extracted and transcriptional changes in genes of the fimsbactins locus were assessed by qRT-PCR and normalized to the expression of *rpoB*. Expression of *bfnL* was used as a negative control. * p < 0.05, ** p < 0.01 and **** p < 0.0001, as determined by Student’s *t* test relative to a hypothetical value of 1. Data are representative of two experiments performed in biological quadruplicate.
(TIFF)

S7 Fig Siderophore production impacts the growth kinetics of *A*. *baumannii* and is required for growth on human serum and transferrin as sole iron sources. Growth kinetics of WT *A*. *baumannii* ATCC 17978 and its isogenic siderophore biosynthetic mutants, as indicated, were analyzed from the data presented in Fig 7. Estimates of the maximal OD_600_ (asymptote (A) A-D), maximum specific growth rate (μ_m_, E-H) and lag time (λ, I-L) are given for the conditions listed where are given where *p < 0.05, ** p < 0.01, *** p < 0.001, and **** p < 0.0001. When growth was insufficient to calculate the parameter, no data is given (ND).
(TIFF)

S8 Fig Metal responsive genes are upregulated in the *A*. *baumannii* infected host. Mice were systemically infected with WT *A*. *baumannii* and sacrificed at 24 h post-infection. Organs were harvested, RNA extracted, and gene expression changes relative to growth *in vitro* were determined in the kidney (A), liver (B), and spleen (C) using NanoString technology. Genes are clustered by known or predicted function, as indicated.
(TIFF)
