# Supplementary material for: Acinetobacter baumannii can use multiple siderophores for iron acquisition, but only acinetobactin is required for virulence
Source: PLoS Pathog. 2020 Oct 19;16(10):e1008995. doi: 10.1371/journal.ppat.1008995 (PMC7595644; doi:10.1371/journal.ppat.1008995)
Supplement: S1 Table — (DOCX) [file ppat.1008995.s001.docx]

**S1 Table – Genes involved in acinetobactin biosynthesis and utilization in *A. baumannii* ATCC 17978**

| **Acinetobactin biosynthesis and transport** | | |
| --- | --- | --- |
| **^a^Locus** | **Gene name** | **Known or proposed function** |
| A1S_2392 | *bauF* | Putative acinetobactin utilization protein; NADPH-dependent ferric siderophore reductase |
| A1S_2391 | *basA* | Putative acinetobactin biosynthesis protein |
| A1S_2390 | *basB* | Putative acinetobactin biosynthesis protein |
| A1S_2389 | *bauD* | Putative ferric acinetobactin uptake system; permease protein |
| A1S_2388 | *bauC* | Putative ferric acinetobactin uptake system; permease protein |
| A1S_2387 | *bauE* | Putative ferric acinetobactin uptake system; ATPase component |
| A1S_2386 | *bauB* | Putative ferric acinetobactin uptake system; periplasmic binding protein |
| A1S_2385 | *bauA* | TonB-dependent siderophore receptor protein |
| A1S_2384 | *basC* | Siderophore biosynthesis protein; putative histamine N-monooxygenase |
| A1S_2383 | *basD* | Siderophore biosynthesis protein; NRPS |
| A1S_2382 | *basD* | Siderophore biosynthesis protein; NRPS |
| A1S_2381 | *^b^basE* | Siderophore biosynthesis protein; enterobactin synthase subunit E (*entE* homologue) |
| A1S_2380 | *^b^basF* | Siderophore biosynthesis protein; isochorismatase (*entB* homologue) |
| A1S_2379 | *basG* | Siderophore biosynthesis protein; histidine decarboxylase |
| A1S_2378 | *barA* | Putative siderophore efflux protein |
| A1S_2377 | *barA* | Putative siderophore efflux protein |
| A1S_2376 | *barA* | Putative siderophore efflux protein |
| A1S_2375 | *barB* | Putative siderophore ABC transporter |
| A1S_2374 | *basH* | Siderophore biosynthesis protein; pimeloyl-ACP methyl ester carboxylesterase |
| A1S_2373 | *basI* | Siderophore biosynthesis protein; 4'-phosphopantetheinyl transferase superfamily |
| A1S_2372 | *^b^basJ* | Siderophore biosynthesis protein; isochorismatase synthetase (*entC* homologue) |

^a^Locus tags are from *A. baumannii* ATCC 17978 NC_009085

^b^Genes with homologues in fimsbactins biosynthetic locus
