# Supplementary material for: Acinetobacter baumannii can use multiple siderophores for iron acquisition, but only acinetobactin is required for virulence
Source: PLoS Pathog. 2020 Oct 19;16(10):e1008995. doi: 10.1371/journal.ppat.1008995 (PMC7595644; doi:10.1371/journal.ppat.1008995)
Supplement: S2 Table — (DOCX) [file ppat.1008995.s002.docx]

**S2 Table – Genes involved in baumannoferrin biosynthesis and utilization in *A. baumannii* ATCC 17978**

| **Baumannoferrin biosynthesis and transport** | | |
| --- | --- | --- |
| **^a^Locus tag** | **Gene name** | **Known or proposed function** |
| A1S_1647 | *bfnA* | Siderophore biosynthesis protein; IucA/IucC |
| A1S_1648 | *bfnB* | Siderophore biosynthesis protein; lysine/ornithine N-monooxygenase |
| A1S_1649 | *bfnC* | Siderophore efflux; MFS drug transporter |
| A1S_1650 | *bfnD* | Siderophore biosynthesis protein; IucA/IucC (AcsC, similar to *sbnF*) |
| A1S_1651 | *bfnD* | Siderophore biosynthesis protein; LucA/IucC (AcsC, similar to *sbnF*) |
| A1S_1652 | *bfnE* | Siderophore biosynthesis protein; IucA/IucC (Similar to *sbnC*) |
| A1S_1653 | *bfnF* | 2Fe-2S binding protein, FhuF/FhuC ferric oxidoreductase protein |
| A1S_1654 | *bfnG* | Siderophore biosynthesis; putative demethylmenaquinone methyltransferase |
| A1S_1655 | *bfnH* | Ferric siderophore receptor protein |
| No locus tag | *bfnI* | Hypothetical siderophore biosynthesis protein |
| A1S_1656 | *bfnJ* | Uncharacterized membrane protein (PiuB); PepSY transmembrane superfamily |
| No locus tag | *bfnK* | Putative metal transport protein |
| A1S_1657 | *bfnL* | Siderophore biosynthesis protein (AlcB); putative acetyltransferase |

^a^Locus tags are from *A. baumannii* ATCC 17978 NC_009085
