# Supplementary material for: Acinetobacter baumannii can use multiple siderophores for iron acquisition, but only acinetobactin is required for virulence
Source: PLoS Pathog. 2020 Oct 19;16(10):e1008995. doi: 10.1371/journal.ppat.1008995 (PMC7595644; doi:10.1371/journal.ppat.1008995)
Supplement: S3 Table — (DOCX) [file ppat.1008995.s003.docx]

**S3 Table – Genes involved in fimsbactins biosynthesis and utilization in *A. baumannii* ATCC 17978**

| **Fimsbactins biosynthesis and transport** | | |
| --- | --- | --- |
| **^a^Locus tag** | **Gene name** | **Known or proposed function** |
| A1S_2582 | *fbsA* | AraC family transcriptional regulator |
| A1S_2581 | *^b^fbsB* | Siderophore biosynthesis protein; isochorismatase synthetase (*entC* homologue) |
| A1S_2580 | *^b^fbsC* | Siderophore biosynthesis protein; isochorismatase (*entB* homologue) |
| A1S_2579 | *fbsD* | Siderophore biosynthesis protein; 2,3-dihydroxybenzoate-2,3-dehydrogenase (*entA* homologue) |
| A1S_2578 | *fbsE* | Siderophore biosynthesis protein; nonribosomal peptide synthase (NRPS) |
| A1S_2577 | *fbsF* | Siderophore biosynthesis protein; NRPS |
| A1S_2576 | *fbsF* | Siderophore biosynthesis protein; NRPS |
| A1S_2575 | *fbsG* | Siderophore biosynthesis protein; NRPS |
| A1S_2574 | *^b^fbsH* | Siderophore biosynthesis protein; 2,3-dihydroxybenzoate-AMP ligase (*entE* homologue) |
| A1S_2573 | *^b^fbsH* | Siderophore biosynthesis protein; 2,3-dihydroxybenzoate-AMP ligase (*entE* homologue) |
| A1S_2572 | *fbsI* | Siderophore biosynthesis protein; putative Lysine/ornithine N-monooxygenase |
| A1S_2571 | *fbsJ* | Siderophore biosynthesis protein; putative ornithine decarboxylase |
| A1S_2570 | *fbsK* | Siderophore biosynthesis protein; putative acetyltransferase |
| A1S_2569 | HP | Hypothetical protein |
| A1S_2568 | *fbsL* | Siderophore biosynthesis protein; phosphopantetheinyltransferase component of enterobactin synthase multienzyme complex |
| A1S_2567 | *fbsM* | Siderophore biosynthesis protein; putative thioesterase |
| A1S_2566 | *fbsN* | TonB-dependent siderophore receptor protein |
| A1S_2565 | *fbsO* | MFS transporter; putative siderophore uptake protein |
| A1S_2564 | *fbsP* | Siderophore interacting protein; putative reductase |
| A1S_2563 | *fbsP* | Siderophore interacting protein; putative reductase |
| A1S_2562 | *fbsQ* | Putative siderophore efflux protein; ABC-type multi-drug/toxic compound extrusion family |

^a^Locus tags are from *A. baumannii* ATCC 17978 NC_009085

^b^Genes with homologues in acinetobactin biosynthetic locus
