# Supplementary figures and images for: Acinetobacter baumannii can use multiple siderophores for iron acquisition, but only acinetobactin is required for virulence
Source: PLoS Pathog. 2020 Oct 19;16(10):e1008995. doi: 10.1371/journal.ppat.1008995 (PMC7595644; doi:10.1371/journal.ppat.1008995)

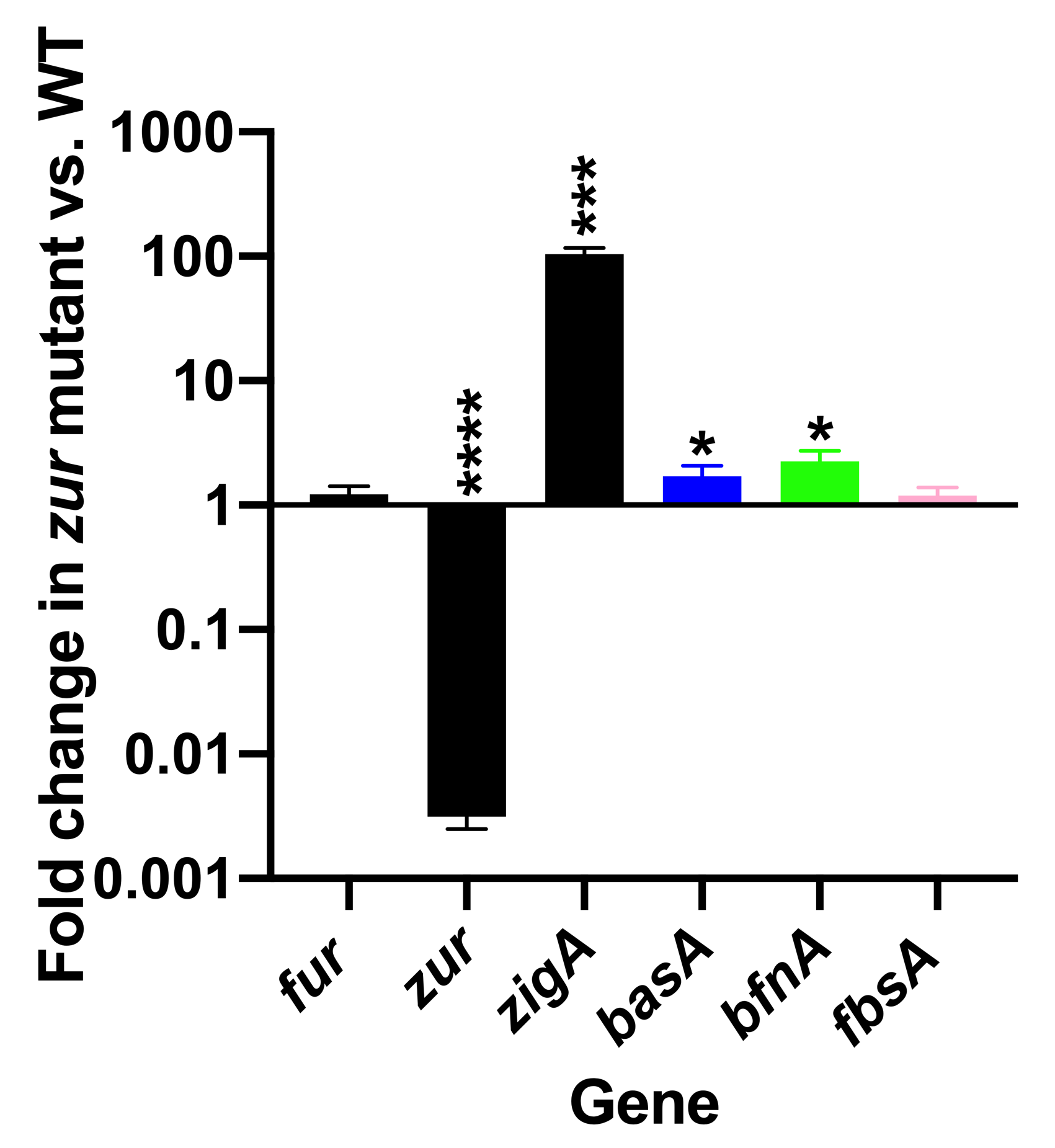

Supplement: S1 Fig — WT A. baumannii and its isogenic Δzur mutant were grown in metal-restricted media for 12 h. RNA was extracted and transcriptional changes in the expression of siderophore-associated genes and fur were assessed by qRT-PCR and normalized to the expression of rpoB. Expression of a known zur-regulated gene, zigA, was assessed as a positive control, whereas zur was run as a negative control. * p < 0.05, *** p < 0.001, and **** p < 0.0001 as determined by Student’s t test relative to a hypothetical value of 1. Data are representative of two experiments performed in biological quadruplicate. (TIFF) [file ppat.1008995.s004.tiff]

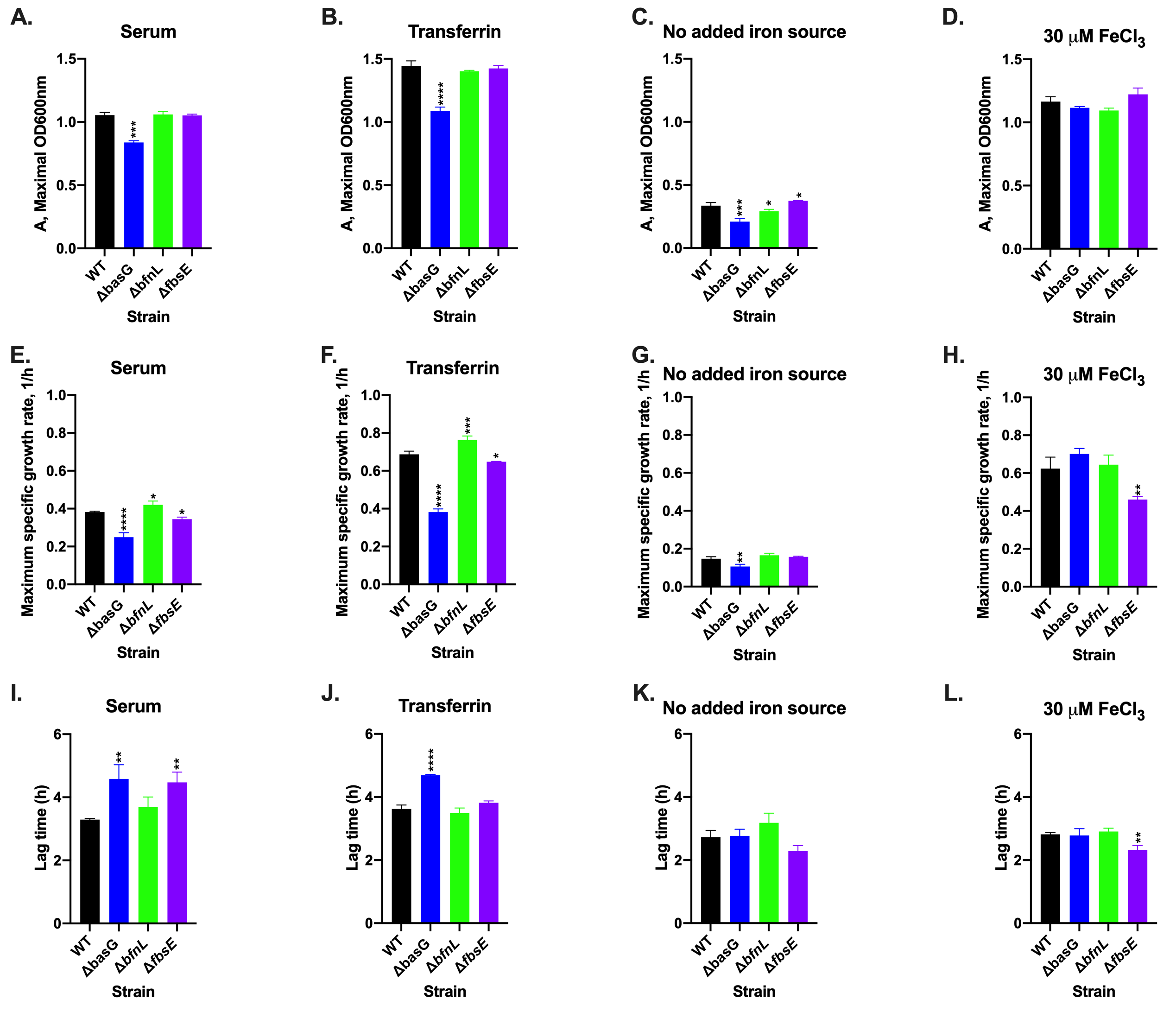

Supplement: S2 Fig — Growth kinetics of WT A. baumannii ATCC 17978 and its isogenic acinetobactin (ΔbasG), baumannoferrin (ΔbfnL) and fimsbactins (ΔfbsE) biosynthetic mutants were analyzed from the data presented in Fig 5. Estimates of the maximal OD600 (asymptote (A) A-D), maximum specific growth rate (μm, E-H) and lag time (λ, I-L) are given where *p < 0.05, ** p < 0.01, *** p < 0.001, and **** p < 0.0001. (TIFF) [file ppat.1008995.s005.tiff]

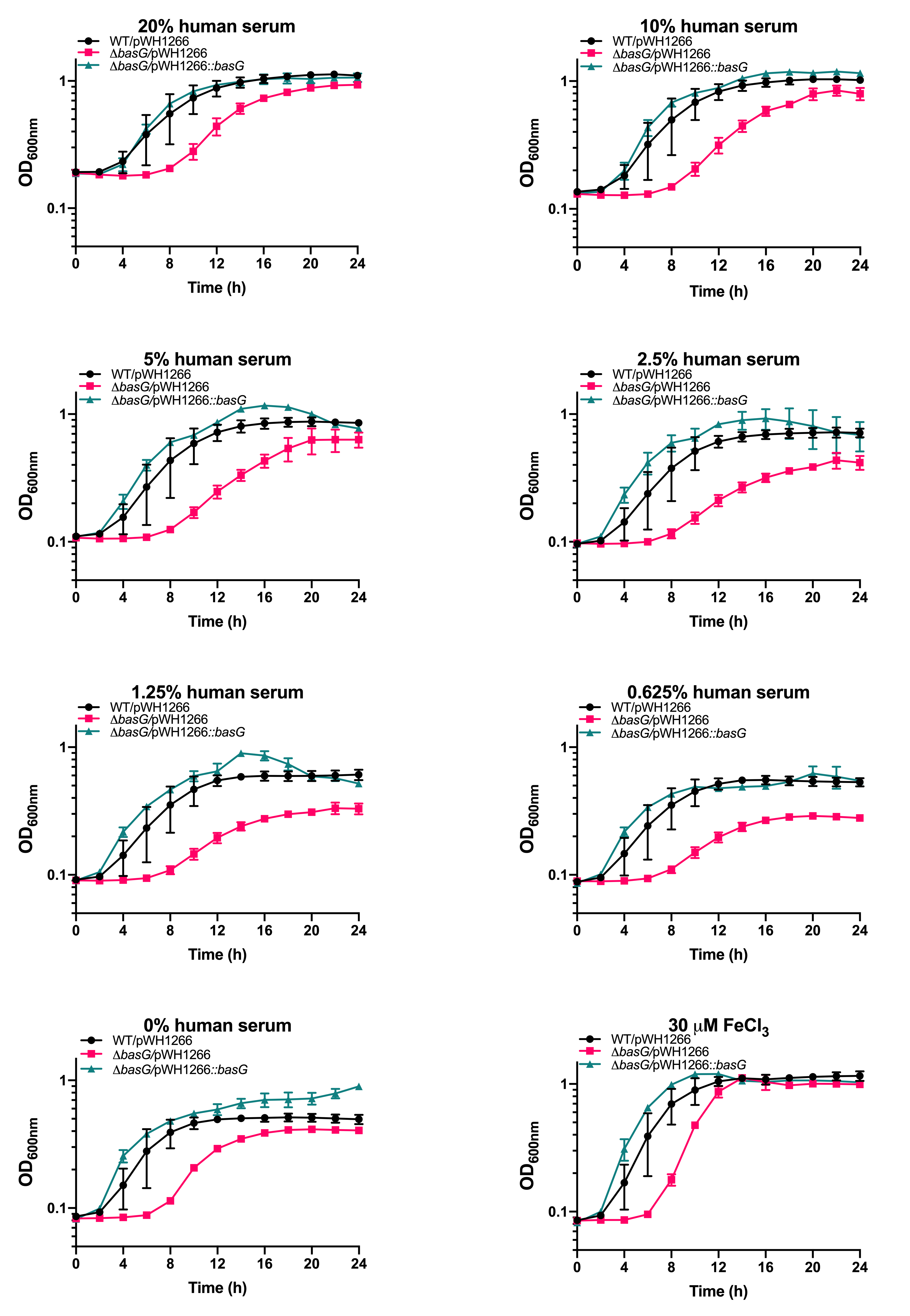

Supplement: S3 Fig — WT A. baumannii ATCC 17978 with empty vector (WT/pWH1266), the acinetobactin-deficient mutant with empty vector (ΔbasG/pWH1266), and a mutant complemented with basG expressed from pWH1266 (ΔbasG/pWH1266::basG) were grown in cTMS media with human serum added at the concentrations indicated. Bacterial growth was assessed by determining the optical density at 600 nm (OD600nm) at the timepoints indicated. Data are the average of technical triplicates and represent the results of two independent experiments. (TIFF) [file ppat.1008995.s006.tiff]

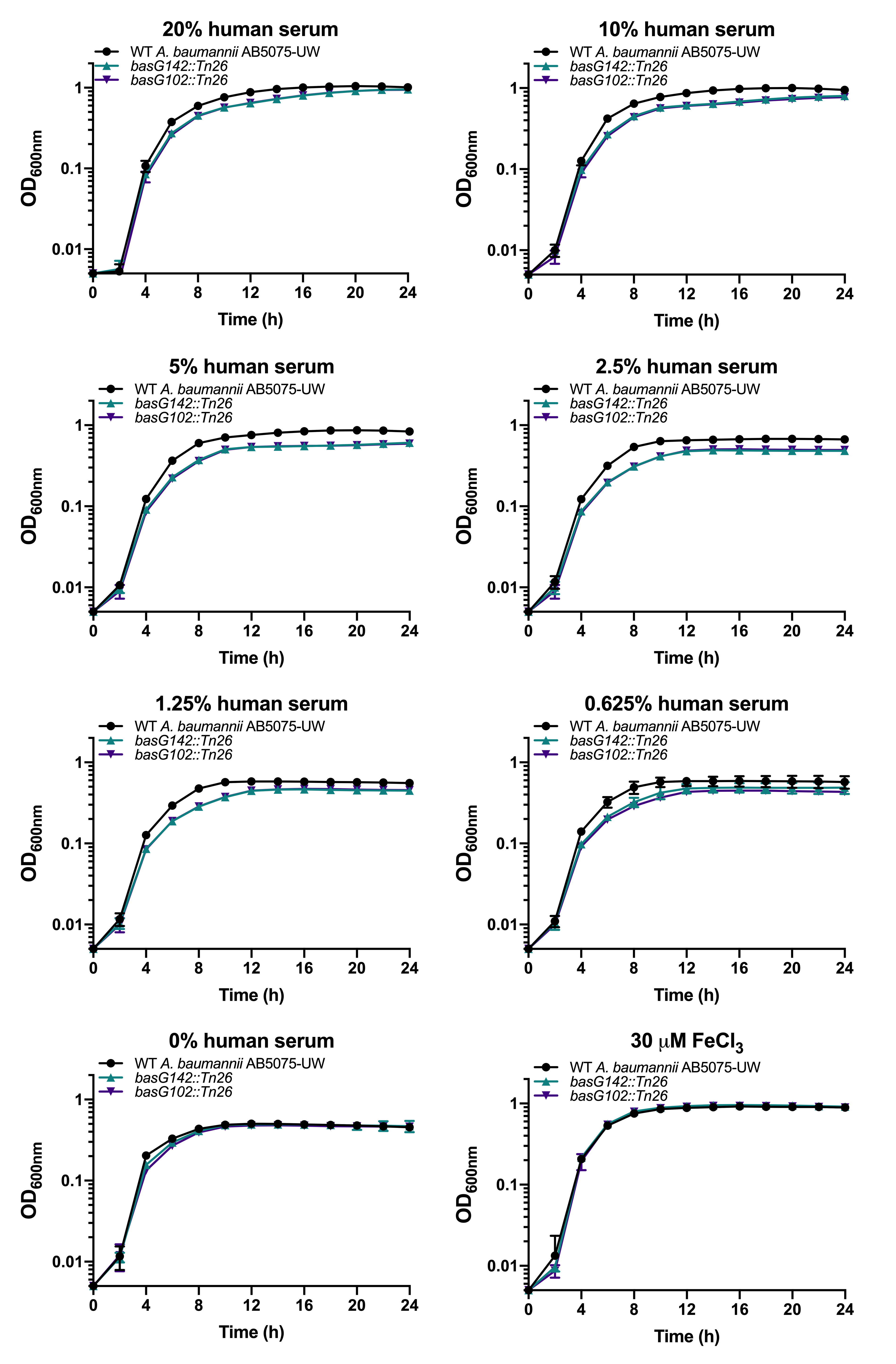

Supplement: S4 Fig — WT A. baumannii AB5075-UW and two unique basG transposon mutants were grown in cTMS media with human serum added at the concentration indicated. Bacterial growth was assessed by determining the OD600nm at the timepoints indicated. Data are the average of technical triplicates and represent the results of two independent experiments. Where error bars are not visible, they are shorter than the height of the symbol. (TIFF) [file ppat.1008995.s007.tiff]

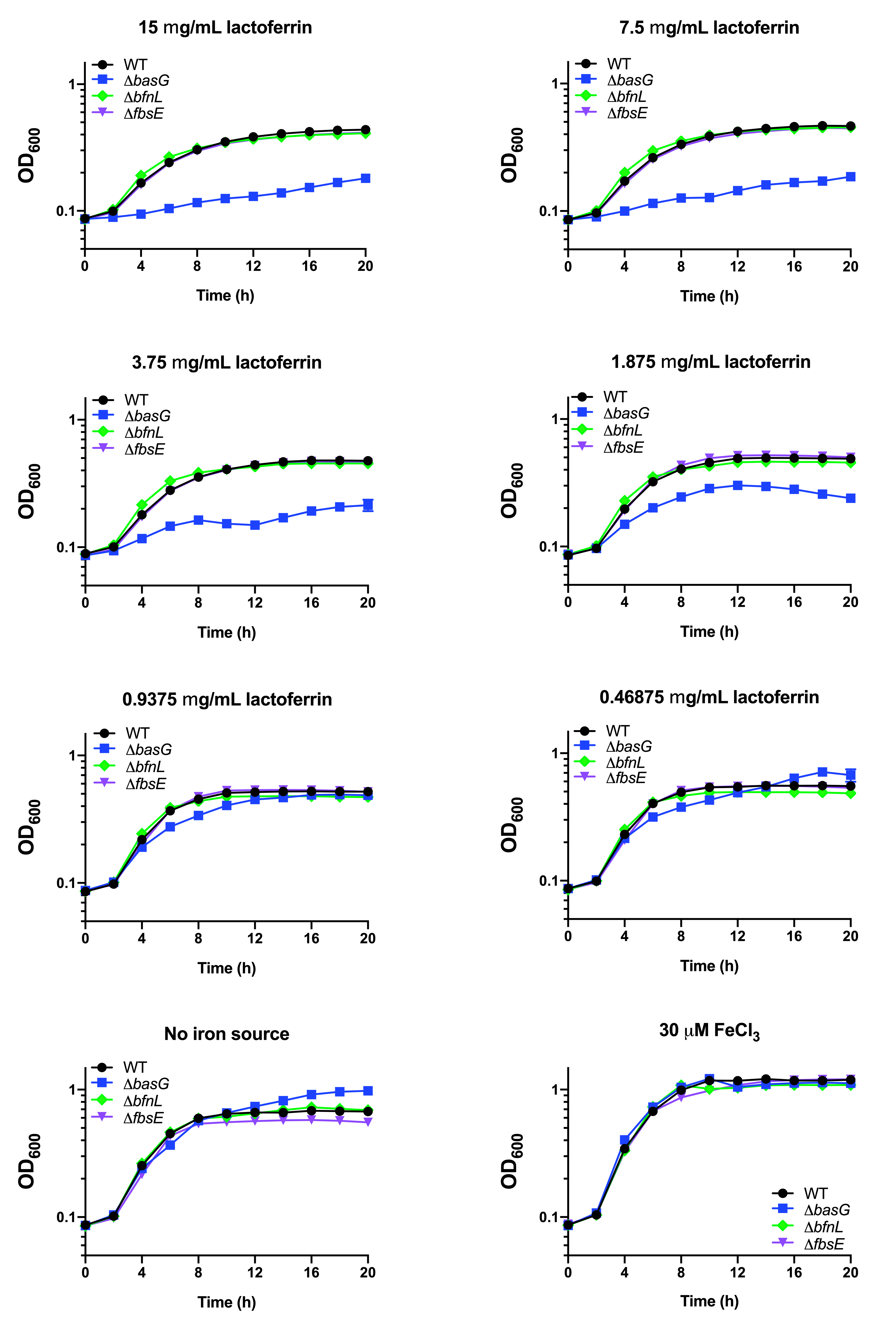

Supplement: S5 Fig — Wild-type (WT) A. baumannii and its isogenic acinetobactin (ΔbasG), baumannoferrin (ΔbfnL) and fimsbactins (ΔfbsE) biosynthetic mutants were grown in cTMS media with lactoferrin, no added iron source, or 30 μM FeCl3, as indicated. Bacterial growth was assessed by determining the OD600nm, at the time points indicated. Data are representative of two independent experiments, and error bars represent the standard error of the mean. Where error bars are not visible, they are shorter than the height of the symbol. (TIFF) [file ppat.1008995.s008.tiff]

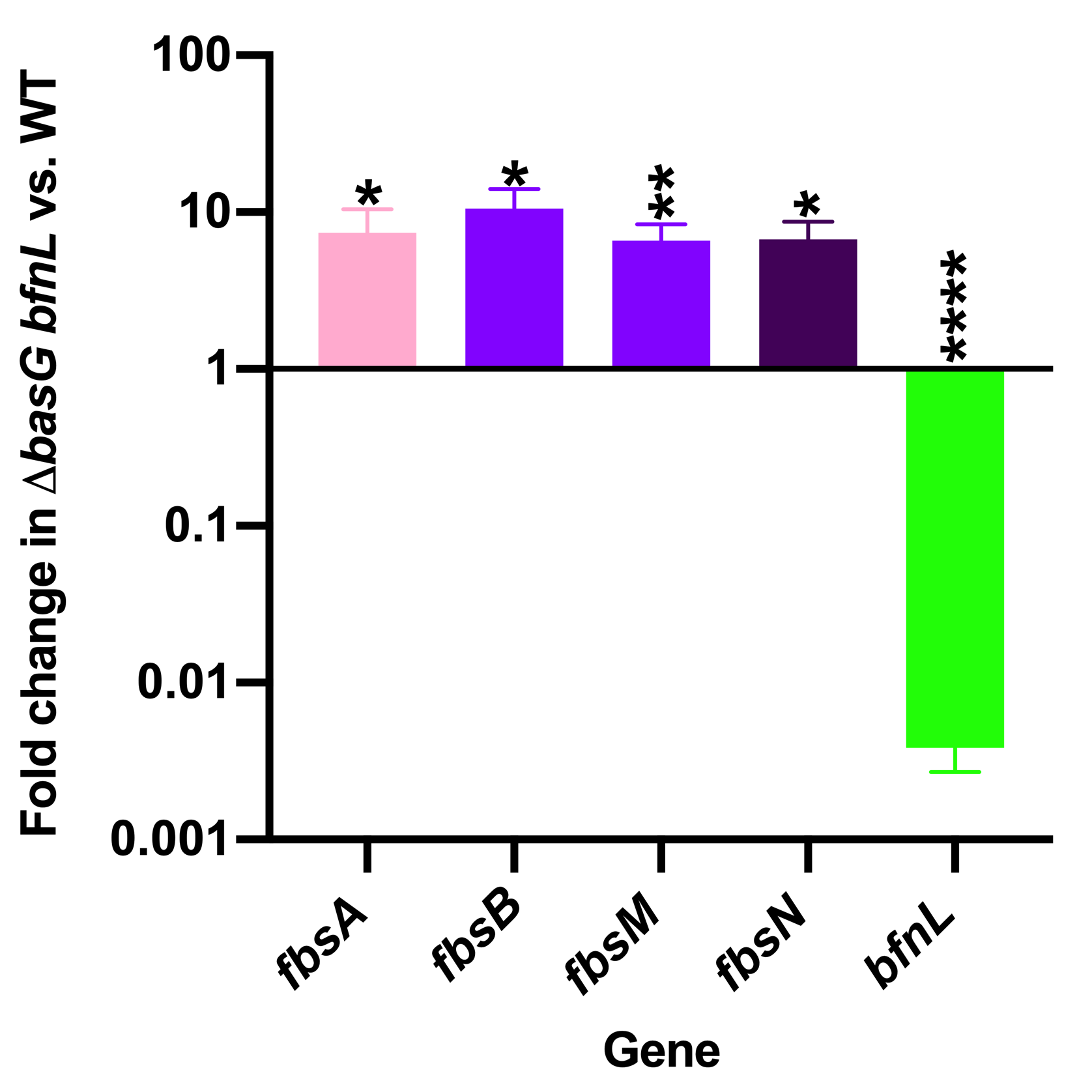

Supplement: S6 Fig — WT A. baumannii and its isogenic ΔbasG bfnL mutant were grown in metal-restricted media for 12 h. RNA was extracted and transcriptional changes in genes of the fimsbactins locus were assessed by qRT-PCR and normalized to the expression of rpoB. Expression of bfnL was used as a negative control. * p < 0.05, ** p < 0.01 and **** p < 0.0001, as determined by Student’s t test relative to a hypothetical value of 1. Data are representative of two experiments performed in biological quadruplicate. (TIFF) [file ppat.1008995.s009.tiff]

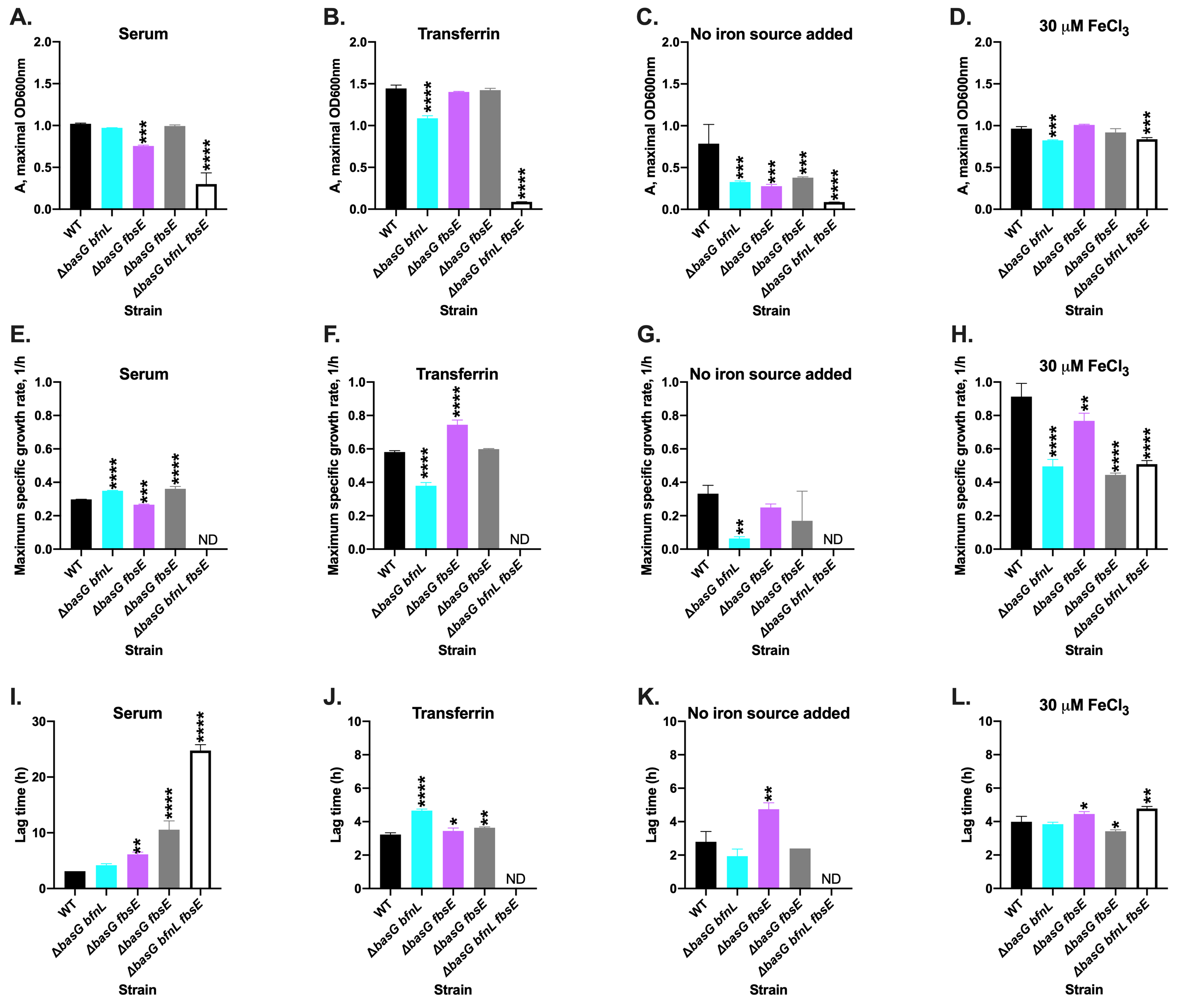

Supplement: S7 Fig — Growth kinetics of WT A. baumannii ATCC 17978 and its isogenic siderophore biosynthetic mutants, as indicated, were analyzed from the data presented in Fig 7. Estimates of the maximal OD600 (asymptote (A) A-D), maximum specific growth rate (μm, E-H) and lag time (λ, I-L) are given for the conditions listed where are given where *p < 0.05, ** p < 0.01, *** p < 0.001, and **** p < 0.0001. When growth was insufficient to calculate the parameter, no data is given (ND). (TIFF) [file ppat.1008995.s010.tiff]

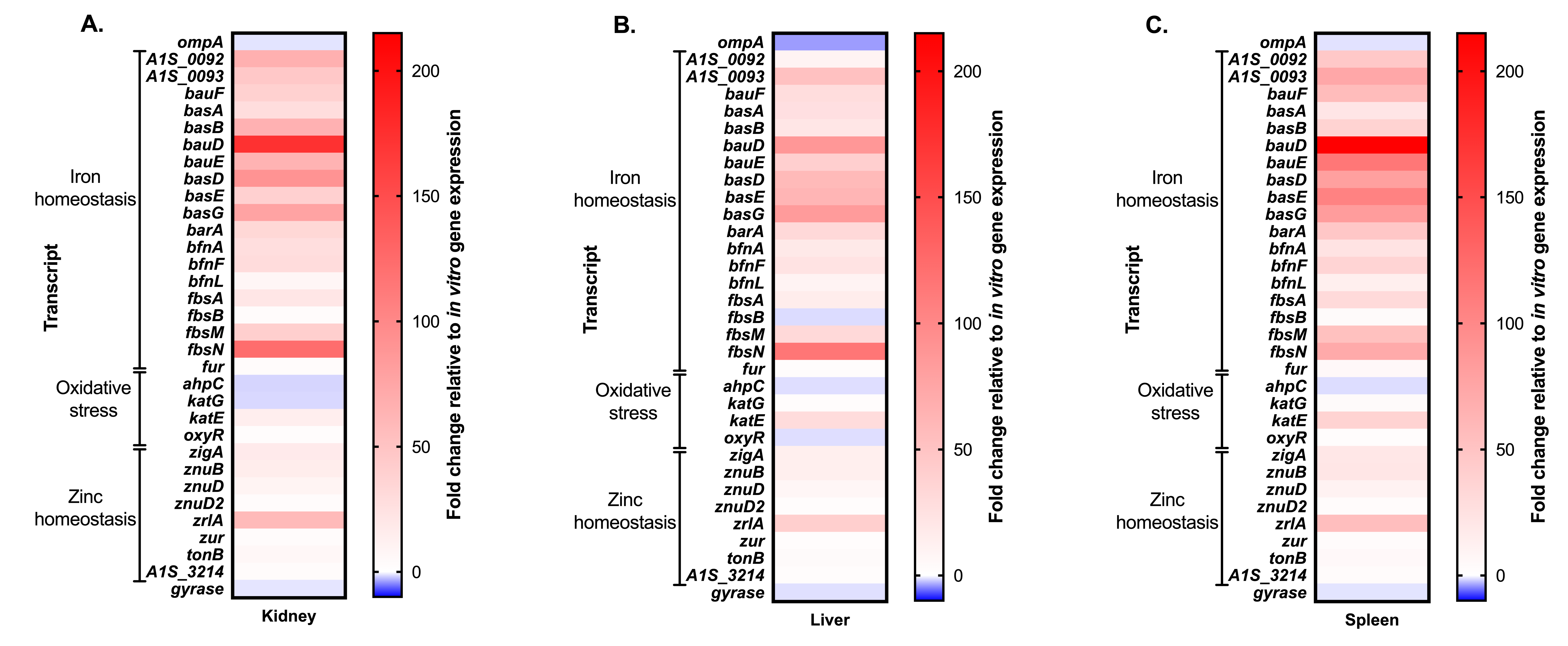

Supplement: S8 Fig — Organs were harvested, RNA extracted, and gene expression changes relative to growth in vitro were determined in the kidney (A), liver (B), and spleen (C) using NanoString technology. Genes are clustered by known or predicted function, as indicated. (TIFF) [file ppat.1008995.s011.tiff]
